# Gremlin-2 is a BMP antagonist that is regulated by the circadian clock

**DOI:** 10.1038/srep05183

**Published:** 2014-06-05

**Authors:** Ching-Yan Chloé Yeung, Nicole Gossan, Yinhui Lu, Alun Hughes, James J. Hensman, Monika L. Bayer, Michael Kjær, Karl E. Kadler, Qing-Jun Meng

**Affiliations:** 1 Wellcome Trust Centre for Cell-Matrix Research, University of Manchester, Oxford Road, Manchester M13 9PT, United Kingdom; 2 Faculty of Life Sciences, University of Manchester, Oxford Road, Manchester M13 9PT, United Kingdom; 3 Department of Computer Science, University of Sheffield, Sheffield S1 4DP, United Kingdom; 4 Institute of Sports Medicine, Department of Orthopedic Surgery M, Bispebjerg Hospital, and Center for Healthy Aging, Faculty of Health Sciences, University of Copenhagen, 2400 Copenhagen, Denmark

## Abstract

Tendons are prominent members of the family of fibrous connective tissues (FCTs), which collectively are the most abundant tissues in vertebrates and have crucial roles in transmitting mechanical force and linking organs. Tendon diseases are among the most common arthropathy disorders; thus knowledge of tendon gene regulation is essential for a complete understanding of FCT biology. Here we show autonomous circadian rhythms in mouse tendon and primary human tenocytes, controlled by an intrinsic molecular circadian clock. Time-series microarrays identified the first circadian transcriptome of murine tendon, revealing that 4.6% of the transcripts (745 genes) are expressed in a circadian manner. One of these genes was *Grem2*, which oscillated in antiphase to BMP signaling. Moreover, recombinant human Gremlin-2 blocked BMP2-induced phosphorylation of Smad1/5 and osteogenic differentiation of human tenocytes *in vitro*. We observed dampened *Grem2* expression, deregulated BMP signaling, and spontaneously calcifying tendons in young *CLOCKΔ19* arrhythmic mice and aged wild-type mice. Thus, disruption of circadian control, through mutations or aging, of *Grem2*/BMP signaling becomes a new focus for the study of calcific tendinopathy, which affects 1-in-5 people over the age of 50 years.

Diseases and injury to tendons are a major biomedical problem. Rotator cuff disease, repetitive motion injuries, complications following iatrogenic injury, and tendon rupture, collectively affect 1-in-3 people over the age of 40 years and incur a significant sociological and economic burden (see^1^,^2^ and references therein). Calcium deposits in the mid-substance of the tendon are common in rotator cuff tendons, Supraspinatus, Achilles and patellar tendons resulting in calcific tendinopathy (CT), which has a prevalence of between 2.7% and 22% depending on age and gender (reviewed by^3^,^4^,^5^,^6^,^7^). Thus, tendinopathies are the most common of the arthropathy disorders. However, the pathophysiology is not always associated with overuse, indicative of an underlying defect in tissue homeostasis as a result of age. An under-researched aspect is the dramatic differences in physiological activity of tendons during a 24-hour cycle of being asleep and awake, and if these differences are influenced by aging and gene expression changes.

The circadian clock coordinates the 24-hourly rhythms in biochemical and behavioral parameters that help prepare an organism for anticipated changes in its environment. In addition to the central pacemaker in the suprachiasmatic nuclei (SCN), many peripheral organs contain autonomous peripheral clocks^8^,^9^. Mammalian circadian clocks rely on the transcriptional/translational feedback loops consisting of the transcriptional activator complex CLOCK and BMAL1 (bone and muscle ARNT-like 1), and repressor complex PERIOD1/2 and CRY1/2. PERs/CRYs periodically inhibit the CLOCK/BMAL1-mediated transactivation of the *Pers* and *Crys* through E-box elements in their promoters. The core oscillator then drives rhythmic clock-controlled genes through regulatory elements in their promoters (e.g. E-box, D-box, and ROREs).

Importantly, mice with a deletion of *Bmal1* show age-related and non-inflammatory arthropathy, including ectopic tendon calcification^10^. It was unknown if the tendon phenotype was the result of *Bmal1* gene-specific effects or to disruptions to the molecular clock, and if the latter, if tendon contains a functional clock that controls tissue homeostasis. Here, we show for the first time the presence of self-sustained circadian clocks in mouse tendon and human tenocytes, and, the circadian clock controls BMP signaling. The results indicate that tendon clocks control 4.6% of local transcripts including *Grem2*, which we show is an antagonist of BMP signaling and inhibits calcification of primary human tenocytes in culture. Disruption of the circadian rhythms in the arrhythmic *CLOCKΔ19 mice*^11^ is associated with aberrant *Grem2* expression and BMP signaling, calcific pathology and mechanically weak tendons. Finally, we show that aged wild-type mice exhibit a dampened and delayed tendon circadian rhythm associated with profound calcification.

## Results

### Autonomous circadian rhythm in tendon

**Figure 1:**
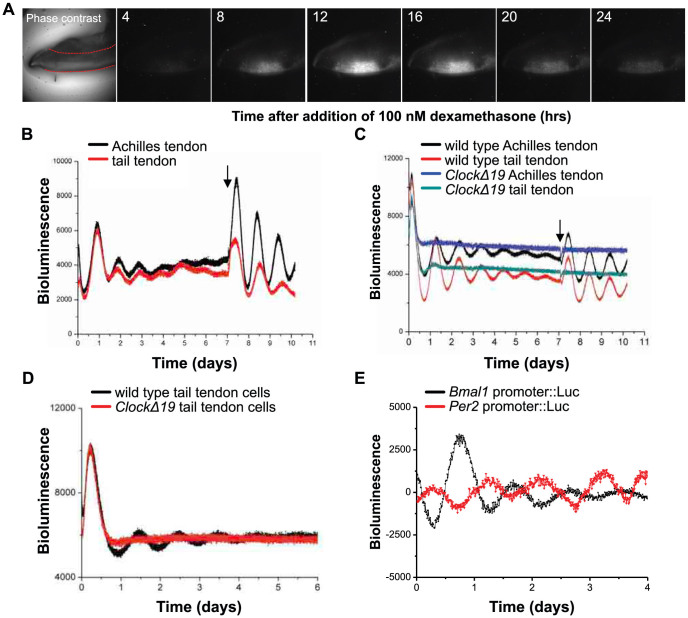
Tendon tissues and cells have an autonomous circadian rhythm. (A) Bioluminescence microscopy of dissected Achilles tendon from PER2::Luc reporter mouse imaged in the presence of 100 nM dexamethasone. Image of the dissected Achilles tendon under phase contrast microscopy is shown, where dotted red lines outline the tendon shaft. (B–D) PMT recordings of endogenous circadian rhythms and re-initiation of the rhythms with 100 nM dexamethasone (arrow) in dissected Achilles and tail tendons from PER2::Luc mice (B); compared with tendons from CLOCKΔ19 mice bred on a PER2::Luc background (C); and in cultured tail tendon cells isolated from wild type and CLOCKΔ19 x PER2::Luc mice (D). (E) PMT recordings of Per2::Luc and Bmal1::Luc reporters expressed in primary human tendon cells. Representative readings from one preparation of cells are shown. Arrow indicates re-initiation of rhythm with 100 nM dexamethasone.

To investigate if tendon has an intrinsic clock we examined Achilles and tail tendons from PER2::Luc reporter mice^12^ and used real-time bioluminescence microscopy and a photomultiplier tube (PMT) to record light emission. This revealed robust circadian rhythms of PER2::Luc activity, with periods of 23.73 ± 0.26 hr and 23.23 ± 0.06 hr in Achilles and tail tendons, respectively, indicating that there is a circadian clock in Achilles and tail tendon (Figure 1A and B, and Supplementary video). As expected, the rhythm dampened over time in culture but was effectively reinstated after a single treatment with dexamethasone, a known synchronizing agent for peripheral clocks.

We next examined the circadian rhythm of Achilles and tail tendons from the CLOCKΔ19 x PER2::Luc crosses. The CLOCKΔ19 mice harbor a deletion in exon 19 of the CLOCK gene producing a dominant negative mutant protein^11^. The results confirmed that CLOCKΔ19 tendons are arrhythmic (Figure 1C). We also established that tendon cells isolated from wild type but not CLOCKΔ19 tail tendons have an autonomous circadian rhythm (Figure 1D). To extend these studies to human tendon cells, primary human tenocytes were transduced with lentivirus carrying *Bmal1*- or *Per2*-promoter-driven luciferase reporters. Both reporters showed circadian oscillation in anti-phase to one another (Figure 1E).

### The circadian transcriptome in tendon

**Figure 2:**
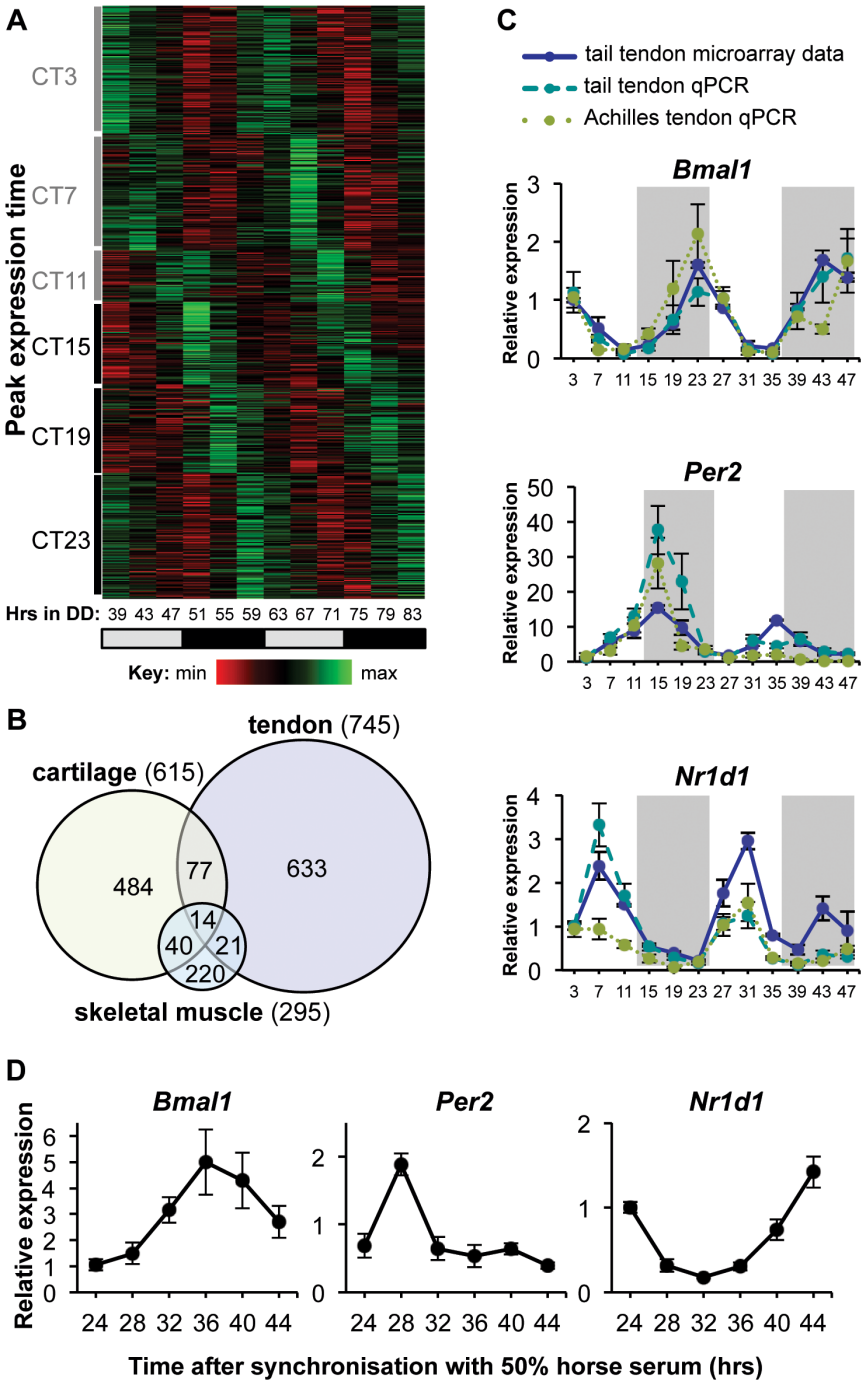
Circadian transcriptome in tendon. (A) Heat map depicting the expression level of the 745 circadian genes (4.6% of the tendon transcriptome) identified by Circwave Batch and JTKCycle. Genes are organized according to timing of peak expression in circadian time (CT). DD = hours in dark/dark cycle. Grey bars represent subjective day; black bars represent subjective night. (B) Specificity of tendon clock genes. Venn diagram comparing the number of circadian genes of tendon, cartilage^9^ and skeletal muscle^8^. The total number of genes identified as circadian in each tissue is represented in brackets; areas of overlap indicate common genes. (C) qPCR validation of time-dependent expression of clock genes, *Bmal1*, *Per2*, and *Nr1d1* (*REV-ERBα*) in mouse tendons. qPCR values (n = 6 for tail tendons; n = 4 for Achilles tendons) normalized to *GAPDH*. Microarray data of tail tendon (n = 2) was plotted for comparison. Grey shadow indicates subjective night phase. (D) Time-dependent expression of clock genes, *Bmal1*, *Per2* and *Nr1d1* (*REV-ERBα*) in primary human tendon cells (n = 3). Cells were synchronized with 50% horse serum and gene expression was analyzed by qPCR. qPCR values normalized to *GAPDH*. Mean data from three preparations of cells shown. Error bars indicate SEM.

Circadian transcriptome studies in muscle, liver and cartilage have demonstrated very low numbers of overlapping rhythmic genes, highlighting the tissue-specific functions of the peripheral circadian clocks (for discussion see^9^). To gain further insights into the functions of the tendon clock, we performed a time-series microarray to identify the circadianly-regulated genes. Female C57BL/6 mice were kept in constant darkness for 39 hr to reveal genes driven by the endogenous circadian clock rather than by light-related cues. Tail tendon was taken at 4-hr intervals during 48 hr. In total, 16,000 genes (represented by 29,000 probes) were classified as expressed. To identify circadian transcripts, we used two well-recognized algorithms based on different statistical models as described previously^9^. CircWave Batch revealed ~1000 probes oscillating with a period of ~24 hr (Figure 2A and Figure S1, q < 0.003). The JTKcycle algorithm identified 1,500 rhythmic probes with 20–28 hr period (q < 0.1). To be stringent, we considered only those genes identified by both methods to be circadianly regulated. From these analyses we identified 745 genes (4.6% of genes expressed in tendon) with rhythmic expression and a period of ~24 hr (Figure 2A). The uniqueness of the tendon circadian transcriptome is shown schematically in Figure 2B in that only 14 genes are common between previously published circadian-regulated genes in cartilage and skeletal muscle^8^,^9^. Among the 14 common genes were the core clock components *Bmal1*, *Per2* and *Nr1d1*, whose circadian expression rhythms were validated by qPCR in mouse Achilles and tail tendon (Figure 2C). Using a Gaussian process model with a Bayesian approach^9^, rhythmic tendon genes were clustered into 9 distinct clusters according to their inferred periodic functions (Figure S2, Supplementary Table 2). Most genes peaked during late night and early day (Figure S3). In addition, expression of endogenous core clock genes, *Bmal1*, *Per2* and *Nr1d1* were rhythmically expressed in primary human tenocytes (Figure 2D). Thus, mouse and human tendon cells exhibit a cell autonomous circadian molecular oscillator.

### *Grem2* as a clock controlled gene in tendon

**Figure 3:**
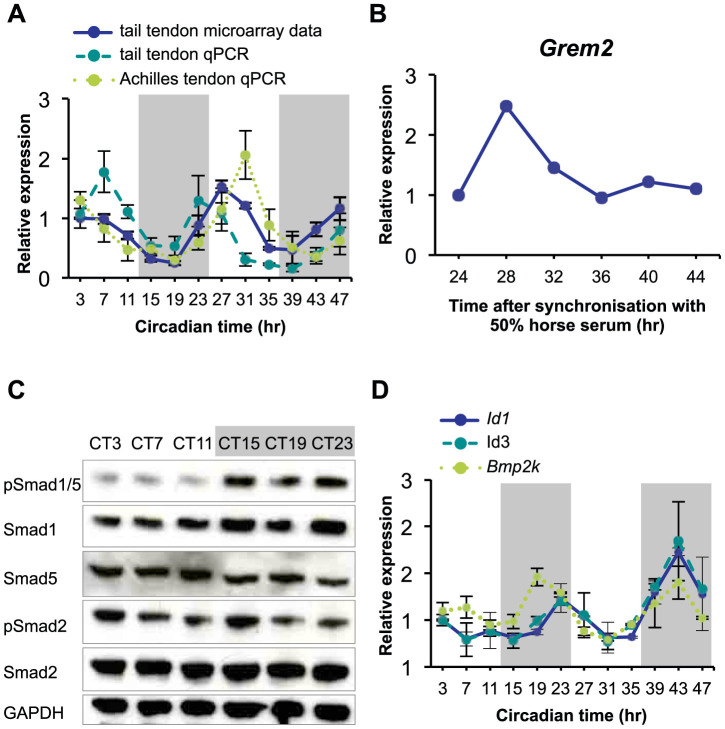
Circadian control of *Grem2* expression and BMP signaling in mouse tendon. (A) Time-dependent expression of *Grem2* from the microarray analysis of tail tendon (n = 2) and qPCR validations in tail (n = 6) and Achilles tendons (n = 4). (B) Time-dependent expression of *Grem2* in primary human tendon cells. Representative data from one preparation of cells shown. Cells were synchronized with 50% horse serum and gene expression was analyzed by qPCR. (C) Wild type mice were kept in DD for 36 hr to allow endogenous circadian rhythm of gene expression. Protein was purified from tail tendons at 4-hr intervals and phosphorylation of Smad1/5 were analyzed by western blotting. Levels of GAPDH protein served as a loading control. Full-length western blots are shown in Supplementary Figure S4. (D) Endogenous expression pattern of BMP signaling target genes, *Id1*, *Id3* and *Bmp2k*, in tail tendon over 44 hr detected by time-series microarray (n = 2). Error bars indicate SEM. Grey shadow indicates subjective night phase.

One of the rhythmic genes in tendon was *Grem2*, with expression peaks in the beginning of day and is down-regulated at night (Figure 3A and B). The *Xenopus* Gremlin is a secreted protein that can block BMP signaling by binding BMPs, thereby preventing receptor activation and phosphorylation of Smad1/5^13^ (reviewed by^14^ and discussed in more detail below). Therefore, we predicted that the mammalian *Grem2* might have a similar role in antagonizing BMP signaling. We used western blot analysis to assess levels of phosphorylated Smads in temporally-collected tail-tendon proteins (Figure 3C). Phosphorylation of Smad1/5 was observed at all 6 time points across one circadian cycle but was noticeably less abundant at CT3, CT7 and CT11 and more abundant at CT15, CT19 and CT23. The total amount of Smad1, Smad5, Smad2 and phosphorylated Smad2 (a known target of TGFβ) did not appear to vary. Target genes of BMP signaling include inhibitor of differentiation 1 (*Id1*), *Id2* and *Id3* and *Bmp2k* (BMP2-inducible kinase)^15^,^16^. The microarray expression profiles of *Id1*, *Id3* and *Bmp2k* were lower in the day phase and higher in the night phase (Figure 3D). Thus, the data support the hypothesis that BMP signaling in tendon is “gated” by the circadian clock, with an opposing pattern to the rhythmic expression of *Grem2*.

### Gremlin-2 inhibits phosphorylation of Smad1/5 and calcium deposition

**Figure 4:**
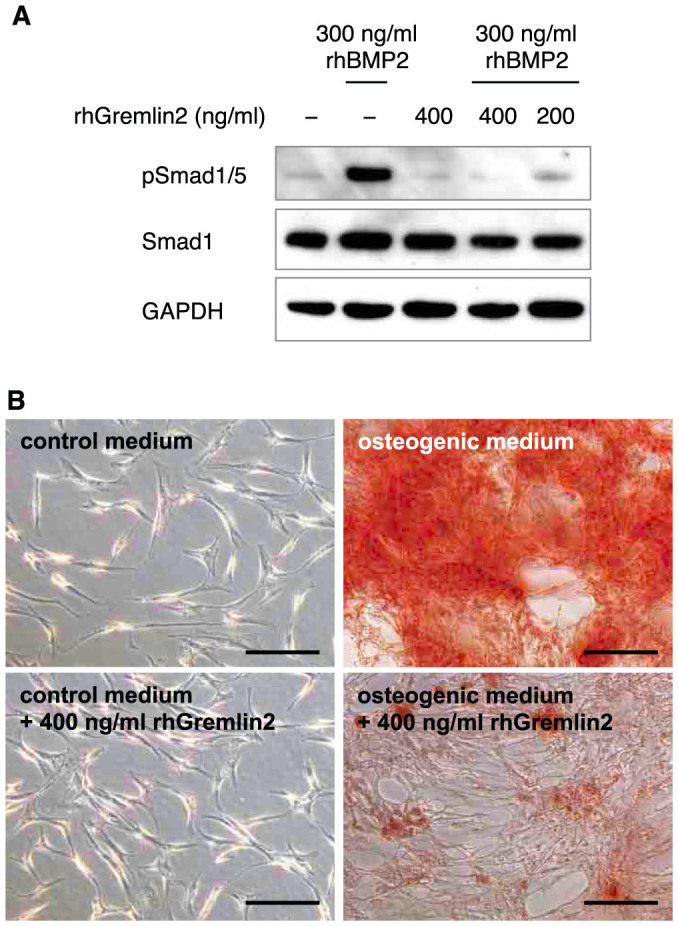
Gremlin-2 inhibits BMP signaling and calcium deposition by human tendon cells. (A) Primary human tendon cells were treated with recombinant human BMP2 in the presence or absence of recombinant human Gremlin-2. Phosphorylation of Smad1/5 was examined by western blotting (n = 3). Western blot analysis of GAPDH protein served as a loading control. Full-length western blots are shown in Supplementary Figure S5. (B) Primary human tendon cells cultured in osteogenic medium in the presence or absence of 400 ng/ml recombinant human Gremlin-2 for 14 days were stained with Alizarin red (n = 3). Bars = 200 µm.

To determine if Gremlin-2 is capable of inhibiting BMP signaling we used an *in vitro* osteogenic differentiation model for human tenocytes. Addition of rhBMP2 to primary human tendon cells activated the phosphorylation of Smad1/5, which was reduced by pre-incubation with rhGremlin-2 (Figure 4A). Next, human tendon cells cultured in an osteogenic medium for 14 days stained bright red by Alizarin red, indicative of calcium deposition (Figure 4B). However, only low-level calcium deposition was observed in cells co-incubated with rhGremlin-2. Together, these data show that Gremlin-2 inhibits BMP signaling and calcium deposition in human tenocytes.

### Aberrant BMP signaling and ectopic calcification in Achilles tendon of *CLOCKΔ19* mice

**Figure 5:**
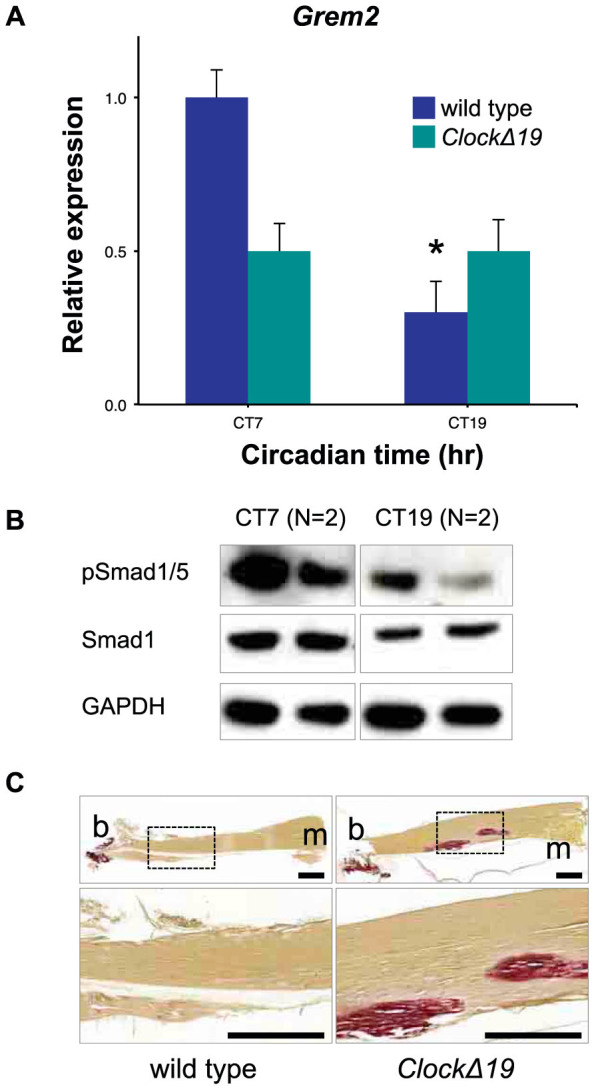
Tendons from CLOCKΔ19 mice exhibit ectopic calcification. (A) Expression of *Grem2* in tail tendons of *CLOCKΔ19* mice compared with wild type tendons at CT7 (peak expression) and CT19 (nadir expression) (n = 3). qPCR values normalized to *GAPDH*. *p < 0.05, one-way ANOVA. Error bars indicate the SEM. (B) Protein was purified from *CLOCKΔ19* tail tendons at CT7 and CT19 and phosphorylation of Smad1/5 were analyzed by western blotting (n = 2). Western blot analysis of GAPDH protein served as a loading control. (C) Longitudinal sections of Achilles tendon from 18 week-old wild type and *CLOCKΔ19* mice stained with Alizarin red. b = attachment of tendon to bone, m = attachment of tendon to muscle. Bars = 500 µm.

The circadian control of *Grem2* and BMP activity prompted us to hypothesize that the CLOCKΔ19 mouse will have aberrant BMP signaling, predisposing the tendons to calcification. To test this, we first compared the expression of *Grem2* in CLOCKΔ19 with wild type tail tendons at two time points corresponding to the peak and trough expression of *Grem2*. Consistent with the loss of circadian rhythms in CLOCKΔ19 tendons (Figure 1C), we observed a lack of time-dependent expression for *Grem2* (Figure 5A). Importantly, at the peak expression time of CT7 in wild type tendons, *Grem2* expression in CLOCKΔ19 tendon was significantly low. The low level of *Grem2* expression was associated with an aberrant pattern of phosphorylated Smad1/5 (Figure 5B). Noteworthy, low level BMP signaling seen in wild-type tendon at CT7 was replaced by elevated BMP signaling in the CLOCKΔ19 tendons, suggesting an inhibitor function of the clock on BMP signaling. Alizarin Red staining of Achilles tendon showed the presence of calcification in tendons of CLOCKΔ19 mice but not in young age-matched wild type mice (Figure 5C).

### Aberrant clock in aged tendons is associated with ectopic calcification

**Figure 6:**
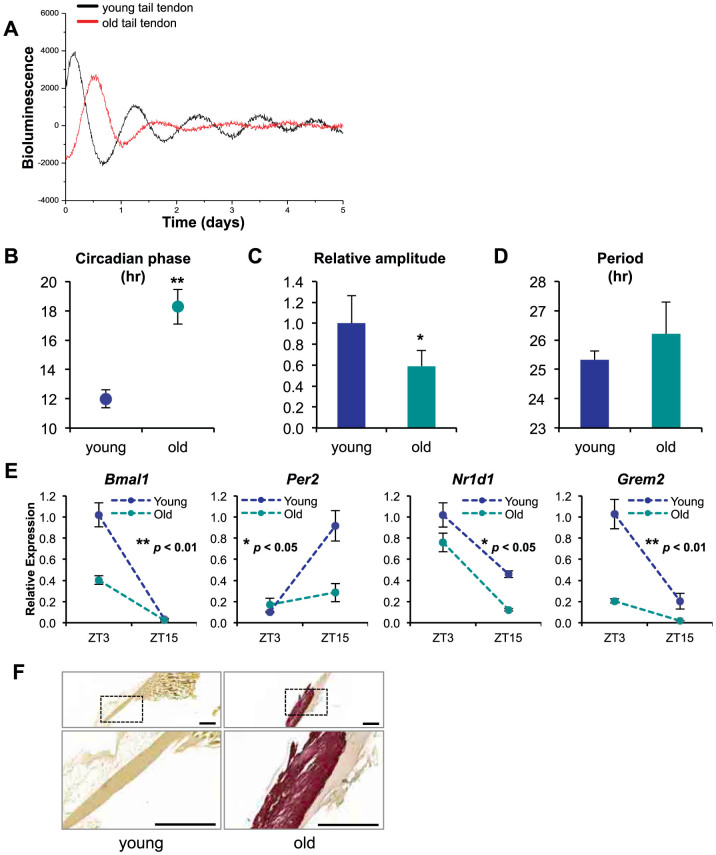
Ectopic calcification in aged tendons correlates with disrupted circadian rhythm and altered *Grem2* expression. (A) Representative PMT recordings (raw and normalized) of ex vivo tail tendons from young (2–3 months) and old (22–24 months) PER2::Luc mice (n = 3). Comparison of (B) circadian phase, (C) amplitude and (D) period between young and aged groups (n = 4). (E) Expression of clock genes, *Bmal1*, *Per2* and *Nr1d1* (REV-ERBα), and *Grem2* in tail tendon of young (2–3 months) and old (22–24 months) at ZT3 and ZT15 (n = 2). qPCR values normalized to *GAPDH*. *p < 0.05, **p < 0.01, unpaired t-tests. Error bars indicate the SEM. (F) Alizarin red staining of longitudinal sections of Achilles tendon from young (2–3 months) and aged (22–24 months) wild type mice. Bars = 500 µm.

To test if the tendon clock changes with ageing, we compared PER2::Luc rhythms in tendon from old (22–24 month old) and young adult (2–3 month old) mice (Fig 6A). There was a significant 40% reduction in the oscillation amplitude of PER2::Luc rhythms in aged tendon and the circadian phase was delayed by ~6 hr (Figure 6B–D). Comparison of the mRNA levels of core clock genes in young and old tendon showed significant dampening in the time-of-day dependent expression of *Bmal1* and *Per2* and reduced levels of *Nr1d1* (Figure 6E). Importantly, in parallel to the clock gene changes, the rhythmic expression of *Grem2* seen in young tendon was severely dampened in the old tendon. Finally, the aged wild-type mice (22–24 month old) developed calcific deposits in their Achilles tendons (Figure 6F).

## Discussion

We have identified cell-autonomous oscillations of clock genes in murine and human tenocytes and shown that the circadian clock is a master regulator of BMP signaling. These results provide a basis for exploring the links between tendon clocks, ageing and tendinopathies.

Circadian clock disruptions have been linked to various pathologies and diseases, including defects in the musculoskeletal system. For instance, bone volume is significantly increased in the *Per2* mutant and *Cry2* knockout mice, caused by defects in osteoblasts and osteoclasts, respectively^17^. Furthermore, shorter bone length was reported in the *Bmal1* knockout mouse because of defects in chondrocyte differentiation in the growth plate^18^. Moreover, maximal muscle force was reduced (by 30%) in the *Bmal1*-null and *CLOCK* mutant mice^19^. Consistently, by comparing the tendon circadian transcriptome to that of the cartilage^9^ and muscle^8^, we found that these tissues had few rhythmic genes in common. In fact, the majority of the common rhythmic genes encoded components of circadian clocks.

Interestingly, the initial phenotypic characterization of the *Mop3* (*Bmal1*) null mouse reported ectopic calcification in tendons and ligaments, among other signs of non-inflammatory arthropathy^10^. The underlying mechanisms for the calcified tendonopathies have never been addressed. Our results showing calcification in the CLOCKΔ19 mouse tendons, support a hypothesis that the CLOCK/BMAL1 complex regulates key signaling pathways that inhibit ectopic calcification in normal rhythmic mice. Thus, when the circadian rhythm is disrupted, the tendons are predisposed to calcification.

BMPs are the largest class of signaling ligands in the TGFβ superfamily and regulate a wide variety of processes during embryo patterning, skeletal and non-skeletal development, differentiation of cell fate, and have additional roles in cancer and tumor progression (reviewed by^20^). The basic signaling mechanism is the same for all TGFβ superfamily members and is initiated when ligand dimers engage with type I and type II serine/threonine kinase receptors. In the activated receptor complex, the type II receptor phosphorylates the type I receptor which provides a binding site for the receptor-regulated SMADs (R-SMADs). The plethora of normal biological processes triggered by BMP signaling, and the involvement of the pathway in pathological conditions including cancer, is partly explained by the large number of BMP ligands (~20 in number), the choice of type I and type II receptors (12 in total) for ligand engagement, and the availability of SMAD dependent and SMAD-independent signal transduction. With such multiple opportunities for pathway activation, the blockade of BMP ligand-receptor engagement by extracellular antagonists is a crucial on-off switch of the signaling pathway. Thus, the circadian clock could be a super-regulator of BMP signaling by regulating the expression of *Grem2*.

Gremlins are small, single domain proteins that contain a cysteine-rich fold that occurs in members of the DAN/Cerberus family of secreted BMP antagonists^21^,^22^,^23^,^24^,^25^. Gremlin-1 prevents binding of BMP receptor binding but does not affect the activity of other members of the TGFβ superfamily^13^. Gremlin-2 (also known as Protein Related to Dan and Cerberus, PRDC) is the second member of the Gremlin family and can inhibit ligand signaling by BMP2 and BMP4 but has minimal effects on GDF-9, activin and TGFβ signaling^26^. Previous studies have shown that *Grem2* restricts BMP signaling to the ventral arches and thereby promotes dorsal and intermediate skeletal fates^27^, is necessary for cardiac laterality and atrial differentiation^28^, and can promote the differentiation of stem cells to cardiomyocytes^29^. However, a role for *Grem2* in tendon homeostasis and calcification had not been previously described. BMP signaling has been shown to drive osteogenic differentiation of tendon-derived stem cells^30^. Furthermore, BMP2 and BMP7 can be detected in the subacromial bursa of patients with chronic rotator cuff degeneration at levels sufficient to induce osteogenic differentiation of mouse myoblast-derived C2C12 cells^31^.

Analysis of our microarray data showed that *Grem2* (but not *Bmp2*, *Bmp4* and *Bmp7*) was rhythmically expressed in tendon. The rhythmic expression of *Grem2* was validated in mouse Achilles and tail tendon and primary human tendon cells. In further experiments we showed that at the peak expression time of CT7, *Grem2* expression in CLOCKΔ19 mutant tail and Achilles tendons was significantly lower than in wild-type tendons, thus indicating a role of the molecular circadian clock in regulating *Grem2* expression in tendon. We also showed that BMP-signaling target genes (*Id1*, *Id3* and *Bmp2k*) are rhythmic, indicating circadian activity of the BMP signaling. A mechanistic link between Gremlin-2 and tendon calcification was evident when Gremlin-2 was capable of inhibiting BMP-induced calcification of human tenocytes in culture, revealing Gremlin-2 as a novel potent antagonist of BMP signaling in tendon. Consistent with the inhibitory role of Gremlin-2, we observed a profound day/night difference in the Smad1/5 phosphorylation opposing the levels of Grem2 expression. Therefore, the circadian control of *Grem2* is likely to be involved in “gating” the BMP signaling pathway in a rhythmic manner.

It is well established that systemic circadian rhythms dampen with aging as shown by body temperature cycles and hormones (see^32^ and references therein). Likewise, we showed that tendon rhythms with altered phase and reduced oscillation amplitude are a feature of aging mice. The delayed phase implies that there is an internal desynchrony between tendon clocks and the central brain clock, which might disrupt the temporal coordination of the tendon rhythmic genes. Such desynchrony in gene expression is exacerbated by the reduced amplitude, leading to compromised tissue homeostasis and increased risk of pathology. As age is a risk factor for calcific tendinopathies in elderly humans, we envisage a scenario in which chronic circadian misalignment/disruption (e.g. during aging) predisposes tendon tissue to calcific changes. Future studies in humans and the use of conditional mutant mouse models may reveal more information about the tendon clock as an important regulator of tissue physiology and pathology.

## Methods

### Animals

All animal work was approved by The University of Manchester Breeding and Supply Unit and was in accordance with the 1986 Home Office Animal Procedures Act (UK), and following local ethical review.

### Tendon Explant Cultures and Bioluminescence Recording

Tendon cultures from PER2::Luc mice were prepared by dissecting out the Achilles and tail tendons. Tendon explants were cultured on 0.4 µm cell culture inserts (Millipore) and bioluminescence was recorded in real-time using as previously described^33^. Dexamethasone (100 nM) was added to the recording media to re-initiate circadian rhythms. Baseline subtraction, period, phase and amplitude calculation was carried out as previously described^9^. Cultures were also visualized using a self-contained Luminoview LV200 microscope (Olympus) and recorded using a cooled Hamamatsu ImageEM C9100-13 EM-CCD camera. Images were taken every hour for 4 days, and combined in ImageJ.

### Time Series Microarrays and Quantitative PCR

The circadian transcriptome studies in tendon were performed as described before^9^. Tail tendons were collected every 4 hr commencing at circadian time CT3 (3 hr into the light phase), for 48 hr. Tissues (6 samples per time point) were snap frozen in liquid nitrogen. Two samples per time point were used for microarray; the remainder was used for qPCR validation. RNA extractions were carried out using TRIzol reagent (Invitrogen) according to manufacturer's protocol. Mouse430_2 Affymetrix GeneChips were run according to manufacturers' instructions. Raw data were deposited in Array Express (accession number pending). CircWave v5 (by Dr. Roelof Hut, cut-off point of q < 0.003) and JTK-Cycle^34^ (q-value of 0.1) were used to identify circadian transcripts as described previously^9^. Local false discovery rate was calculated using an R package ‘fdrtool'^35^. To be stringent, genes identified using both methods are counted as positive. To model the data for clustering, we used a Gaussian process model^9^. Validation of time-series arrays and gene expression analyses was examined by qPCR (for primers see Supplementary Table 1) as described previously^36^.

### Primary Mouse and Human Tenocytes Culture

Cells from tail tendons were released by treatment with 1000 U/ml bacterial collagenase type 4 (Lorne Laboratories) in 0.25% (wt/vol) trypsin (Invitrogen). Cells were grown at 37oC until 90–100% confluent prior to synchronization and PMT recording. Primary human tenocytes were obtained as described previously^37^. Cells from three preparations were used in the experiments described in this study. For time course experiments, confluent cells were synchronized with DMEM:F12 containing 50% (v/v) horse serum (Invitrogen), L-G and P/S for 2 hr at 37oC. RNA was isolated after 24 hr, at 4-hr intervals for 20 hr. For treatment with recombinant human BMP2 (PHC7145, Invitrogen) and recombinant human Gremlin-2 (SRP4657, Sigma), 90–100% confluent cells were washed with PBS and incubated with serum-free media for 1 hr at 37oC. Then, cells were treated with BMP2 that had been pre-incubated for 1 hr at 4oC with Gremlin-2 in serum-free medium. Protein was isolated after 24 hr and analyzed by western blotting.

### Lentiviral Transduction

Lentiviral transduction of primary human tenocytes was performed using methods previously described^9^.

### Protein Extraction and Western Blotting

Proteins from tail tendons were extracted from frozen tissue with ice-cold cell lysis buffer (20 mM Tris/HCl pH 7.6, 150 mM NaCl, 1 mM ethylene diamine tetra-acetic acid (EDTA), 1% (v/v) Igepal CA-630, 50 mM sodium fluoride) containing complete, mini EDTA-free protease inhibitor cocktail and PhosSTOP phosphatase inhibitor cocktail (Roche). Lysates were cleared by centrifugation at 10 000 × g, at 4oC. Commercially available antibodies used to detect phospho-Smad1/5 (#9516), Smad1 (#6933), Smad5 (#9517), phospho-Smad2 (#3108) and Smad2 (#3103) were purchased from Cell Signaling Technologies. GAPDH was detected using a monoclonal antibody from Sigma (G8795).

### Calcium Deposition Assay and Alizarin Red Staining

Achilles tendons were fixed in 2% (w/v) PFA in PBS for 20 minutes, wax processed. 5–6 µm sections were de-waxed before staining. Calcium deposition in primary human tenocytes was induced by culturing cells in Osteogenic Medium (Lonza) according to the manufacturer's protocol. After 14 days, cells were fixed with 2% (w/v) PFA in PBS for 20 minutes. Fixed cells or tissue sections were washed with distilled water and stained with 2% (w/v) Alizarin Red S, pH 4.2 for 10 min. Washed plates or sections were air-dried and imaged using a Pannoramic slide scanner and Pannoramic Viewer software (3DHISTECH).

### Statistical Analyses

Mean and standard error of the mean were calculated for each data set from at least three biological replicates (indicated by the n in figures). Data was evaluated using two sample t-tests with GraphPad Prism (GraphPad Software). Differences were considered significant at the values of p < 0.05 (*), p < 0.01 (**) or p < 0.0001 (***).

## Author Contributions

The author(s) have made the following declarations about their contributions: Conceived the project: K.E.K. Conceived and designed the experiments: K.E.K., Q.-J.M., C.-Y.C.Y. Performed the experiments: C.-Y.C.Y., N.G., Q.-J.M., Y.L., A.H. Contributed reagents/materials/analysis tools: J.J.H., Q.-J.M., M.L.B., M.K. Wrote the paper: K.E.K., Q.-J.M.

## Supplementary Material

Supplementary Information Supplementary information

Supplementary Information Video
