# Supplementary material for: Gremlin-2 is a BMP antagonist that is regulated by the circadian clock
Source: Sci Rep. 2014 Jun 5;4:5183. doi: 10.1038/srep05183 (PMC4046123; doi:10.1038/srep05183)
Supplement: Supplementary Information [file srep05183-s1.pdf]

Gremlin-2 is a BMP antagonist that is regulated by the circadian clock

Ching-Yan Chloé Yeung[1,2], Nicole Gossan[1,2], Yinhui Lu[1,2], Alun Hughes[2], James J. Hensman[3], Monika L. Bayer[4], Michael Kjær[4], Karl E. Kadler[1,2]* and Qing-Jun Meng[1,2]*

[1]Wellcome Trust Centre for Cell-Matrix Research, [2]Faculty of Life Sciences, University of Manchester, Oxford Road, Manchester M13 9PT, United Kingdom,

[3]Department of Computer Science, University of Sheffield, Sheffield S1 4DP, United Kingdom,

[4]Institute of Sports Medicine, Department of Orthopedic Surgery M, Bispebjerg Hospital, and Center for Healthy Aging, Faculty of Health Sciences, University of Copenhagen, 2400 Copenhagen, Denmark

Supplementary 1

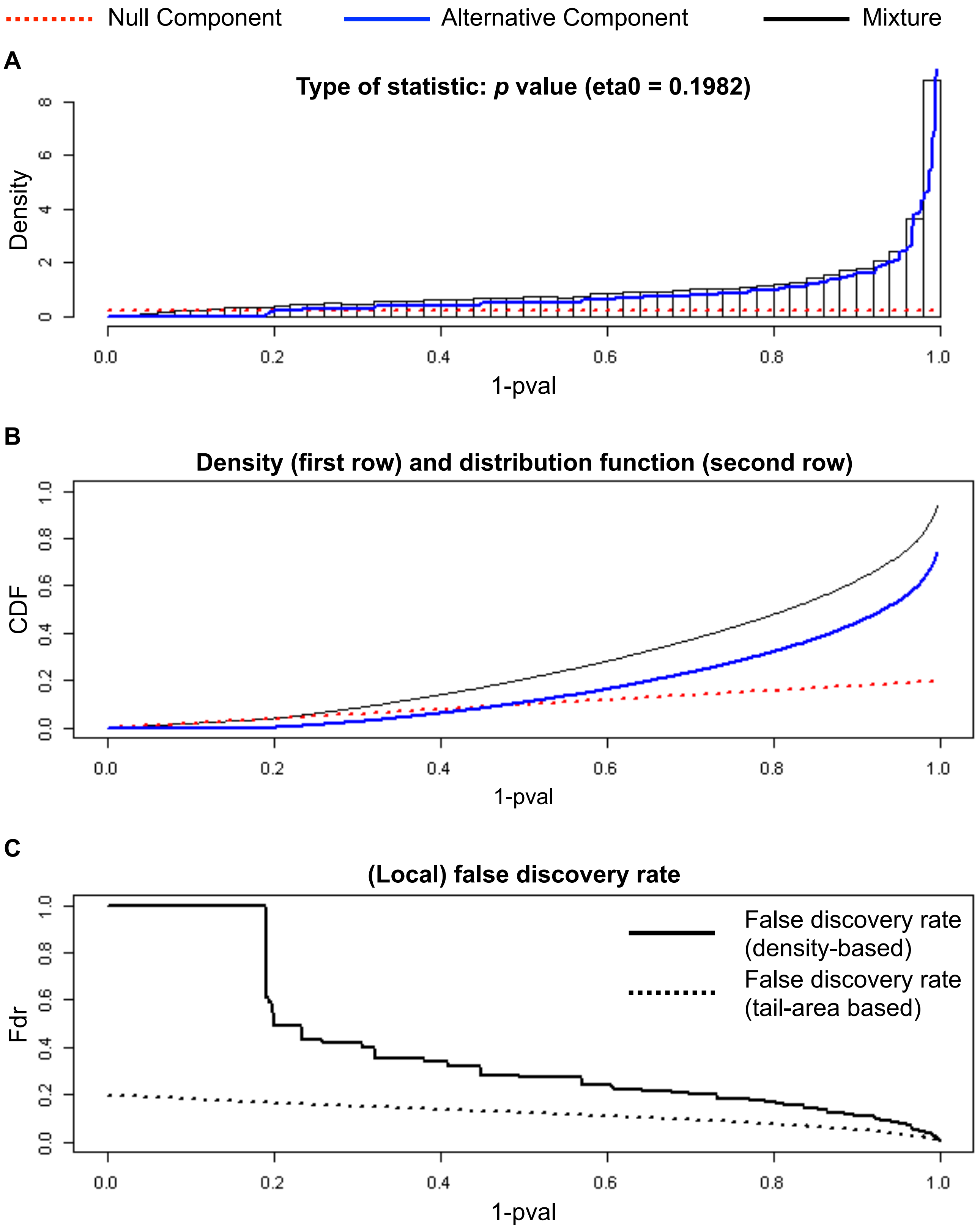

Supplementary 2

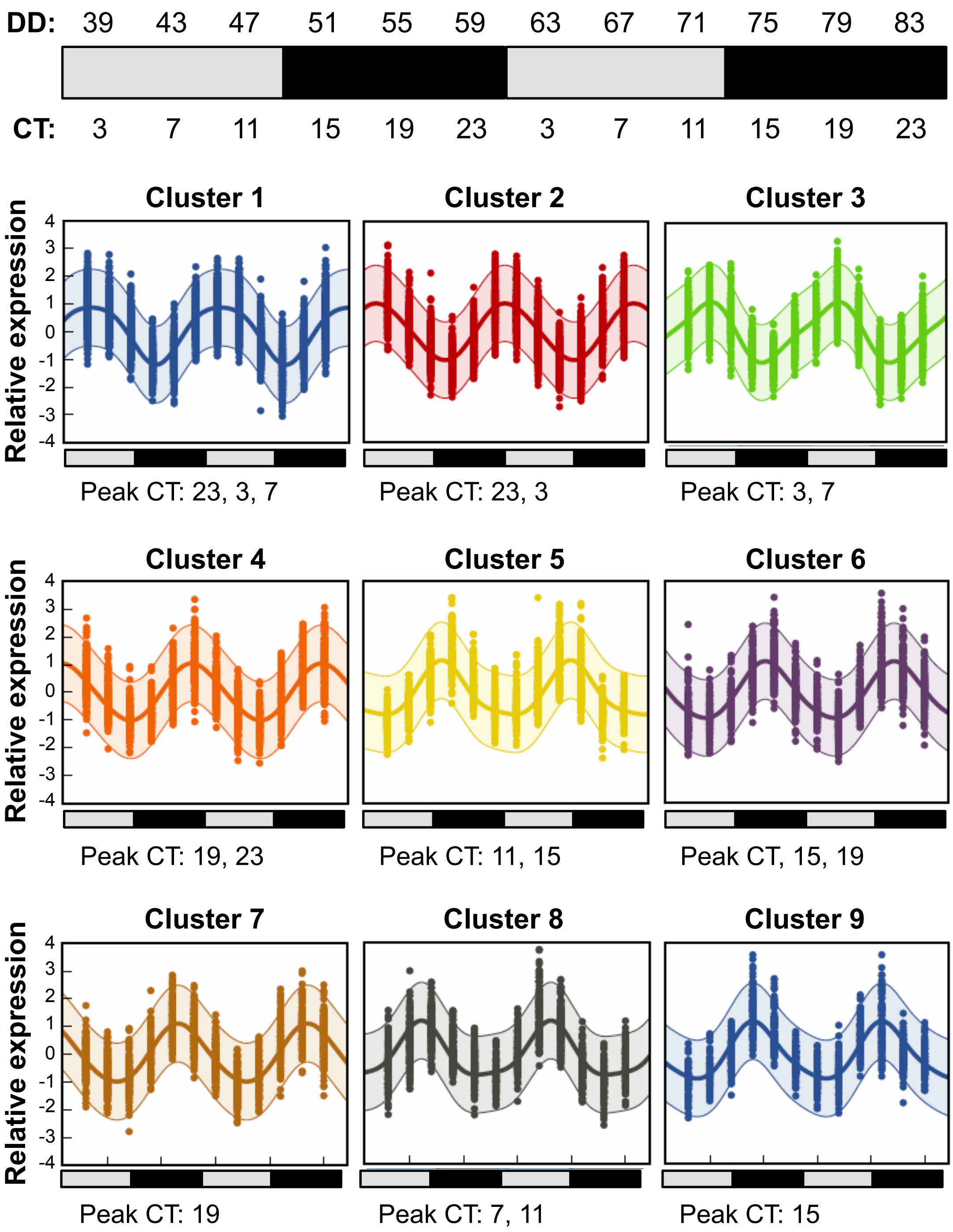

Supplementary 3

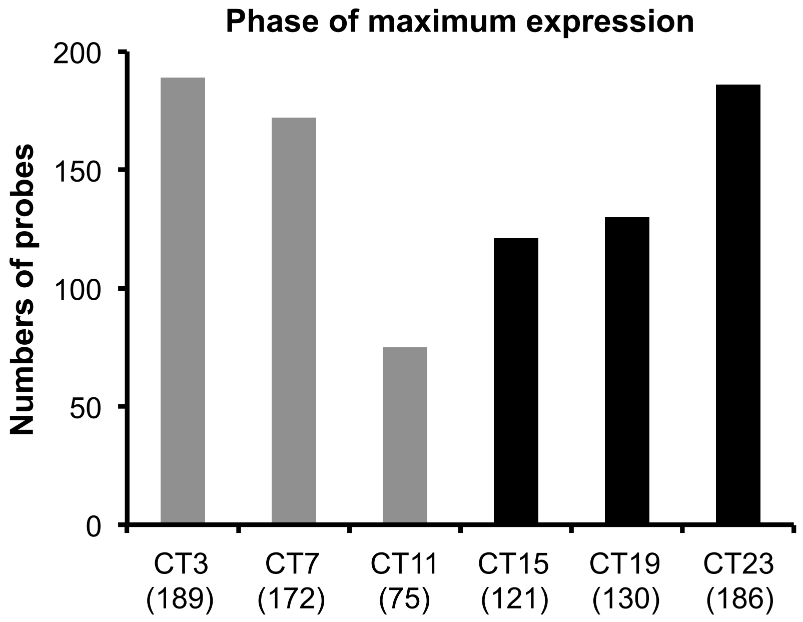

Supplementary Figure 4

pSmad1/5

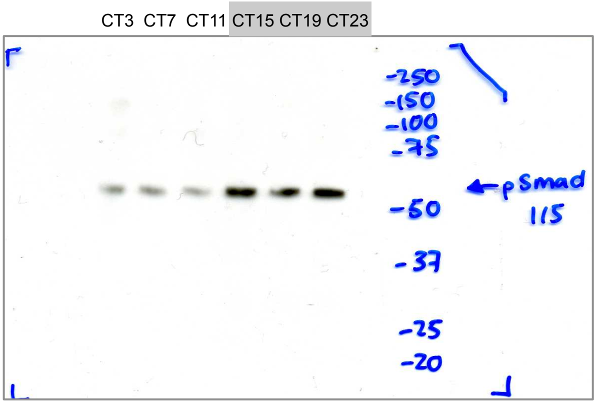

Total Smad1

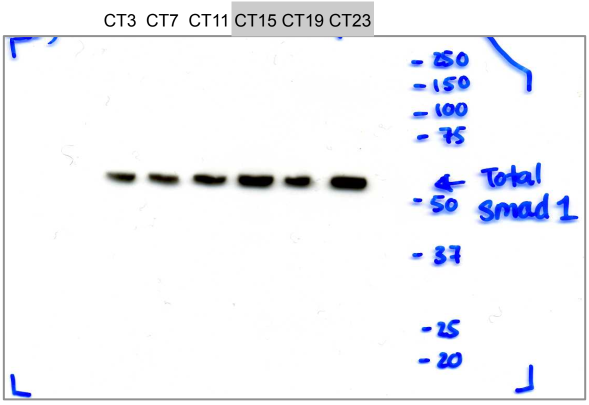

Total Smad5

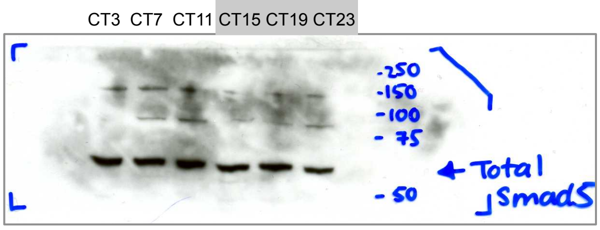

pSmad2

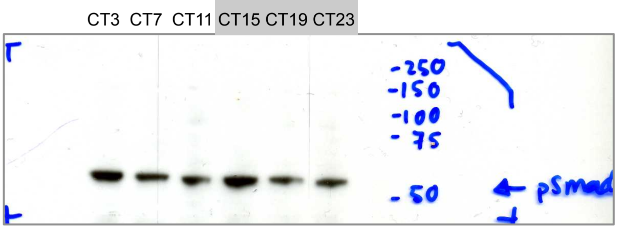

Total Smad2

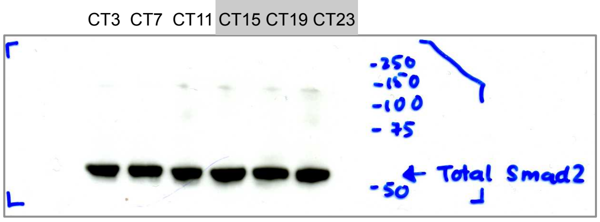

GAPDH

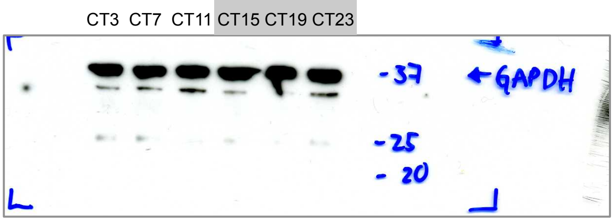

Supplementary Figure 5

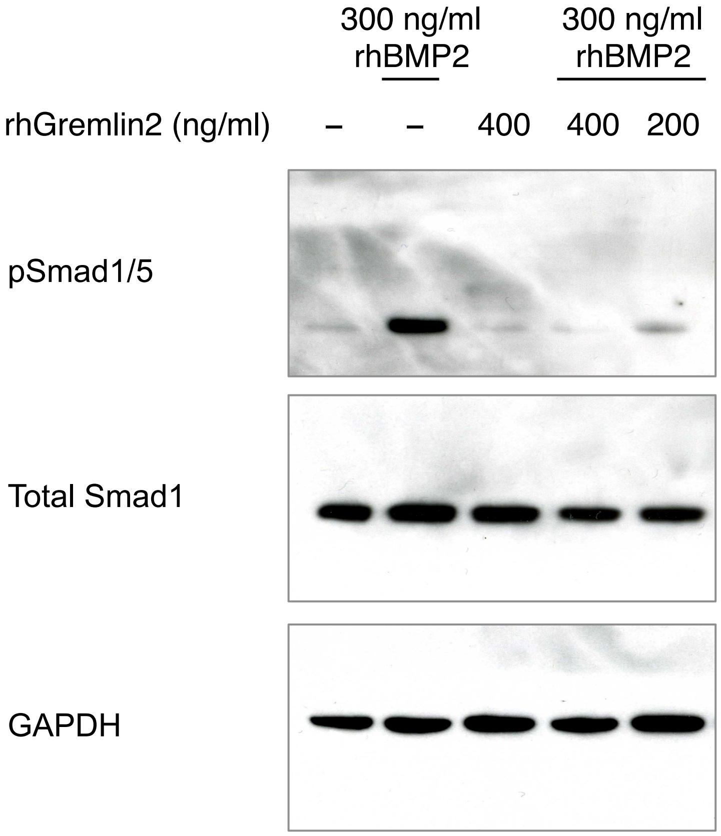

Mouse Gene	Forward primer	Reverse primer	Product size (bp)
Bmal1	5'-CGAAGACAATGAGCCAGACA-3'	5'-AAATAGCTGTCGCCCTCTGA-3'	185
Clock	5'-TGCCAGCTCATGAAAAGATG-3'	5'-CGCTGCTCTAGCTGGTCTTT-3'	199
GAPDH	5'-AACTTTGGCATTGTGGAAGG-3'	5'-ACACATTGGGGGTAGGAACA-3'	223
Grem2	5'-AGGCTTCCATCTCGTCATTG-3'	5'-TGCACCAGTCACTCTTGAGG-3'	244
Nr1d1	5'-CTTCCGTGACCTTTCTCAGC-3'	5'-GGTAAGTGCCAGGGAGTTGA-3'	223
**Human Gene**	**Forward primer**	**Reverse primer**	**Product size (bp)**
Bmal1	5'-TGGATGAAGACAACGAACCA-3'	5'-AGCTGTTGCCCTCTGGTCTA-3'	185
Clock	5'-GGCTGAAAGACGACGAGAAC-3'	5'-GGTGTTGAGGAAGGGTCTGA-3'	210
GAPDH	5'-GAGTCAACGGATTTGGTCGT-3'	5'-GACAAGCTTCCCGTTCTCAG-3'	186
Grem2	5'-TTTCCCTGTCCTTGTTCCTG-3'	5'-TGCACCAGTCACTCTTGAGG-3'	207
Nr1d1	5'-CTGGGAGGATTTCTCCATGA-3'	5'-TCACTGTCTGGTCCTTCACG-3'	182

Supplementary Table 1. Primers used for qPCR.

Supplementary Table 2. List of all rhythmic genes in tendon organized into clusters based on peak expression time.

CLUSTER 1		
**AFFYMETRIX_3PRIME_IVT_ID**	**Name**	**Peak phase**
1418403_at	a disintegrin and metallopeptidase domain 19 (meltrin beta); similar to metalloprotease-disintegrin meltrin beta	3
1434555_at	acidic (leucine-rich) nuclear phosphoprotein 32 family, member A	3
1421918_at	acidic (leucine-rich) nuclear phosphoprotein 32 family, member A	3
1415984_at	acyl-Coenzyme A dehydrogenase, medium chain	3
1435160_at	AHA1, activator of heat shock protein ATPase homolog 2 (yeast)	3
1451083_s_at	alanyl-tRNA synthetase	7
1455673_at	alkB, alkylation repair homolog 2 (E. coli)	3
1436538_at	ankyrin repeat domain 37	3
1428063_at	ankyrin repeat domain 46	3
1423957_at	apoptosis enhancing nuclease	3
1426923_at	ArfGAP with FG repeats 1	23
1455113_at	armadillo repeat containing 8	3
1425914_a_at	armadillo repeat containing, X-linked 1	3
1426403_at	ARP1 actin-related protein 1 homolog B, centractin beta (yeast)	23
1428279_a_at	ataxin 7-like 1	3
1434026_at	ATPase, class I, type 8B, member 2	3
1447679_s_at	BMS1 homolog, ribosome assembly protein (yeast)	7
1439466_s_at	BUD31 homolog (yeast)	3
1418954_at	calcium/calmodulin-dependent protein kinase kinase 1, alpha	3
1455356_at	calmodulin regulated spectrin-associated protein 1; similar to calmodulin regulated spectrin-associated protein 1	23
1431930_x_at	cardiolipin synthase 1	7
1424996_at	CASP8 and FADD-like apoptosis regulator pseudogene; CASP8 and FADD-like apoptosis regulator	3
1418330_at	CCCTC-binding factor	3
1435017_at	CDGSH iron sulfur domain 3	7
1434097_at	cDNA sequence D10627	23
1421963_a_at	cell division cycle 25 homolog B (S. pombe)	3
1460708_s_at	cell division cycle 42 homolog (S. cerevisiae); predicted gene 7407	3
1415724_a_at	cell division cycle 42 homolog (S. cerevisiae); predicted gene 7407	3
1434649_at	cerebral cavernous malformation 2 homolog (human)	3
1442678_at	coiled coil domain containing 28B	3
1418543_s_at	coiled-coil domain containing 43	7
1424831_at	copine II	23
1434923_at	COX19 cytochrome c oxidase assembly homolog (S. cerevisiae)	3
1420827_a_at	cyclin G1	23
1416873_a_at	cyclin-dependent kinase 2	7
1460645_at	cysteine and histidine-rich domain (CHORD)-containing, zinc-binding protein 1	3
1436270_at	cytochrome b5 domain containing 1; predicted gene 6685	3
1429028_at	dedicator of cytokinesis 11	3
1434846_at	DENN/MADD domain containing 4C	23
1415745_a_at	Down syndrome critical region gene 3	3
1418649_at	EGL nine homolog 3 (C. elegans)	3
1415863_at	eukaryotic translation initiation factor 4, gamma 2	3
1434985_a_at	eukaryotic translation initiation factor 4A1	7
1418443_at	exportin 1, CRM1 homolog (yeast)	7
1436185_at	expressed sequence AI314180	7
1428541_at	family with sequence similarity 115, member A	3
1448678_at	family with sequence similarity 118, member A	23
1428353_at	forkhead box K2	3
1434320_at	general transcription factor IIIC, polypeptide 4	23
1448693_at	glycolipid transfer protein domain containing 1	3
1435032_at	golgi autoantigen, golgin subfamily b, macrogolgin 1	3
1418492_at	gremlin 2 homolog, cysteine knot superfamily (Xenopus laevis)	3
1460206_at	GRP1 (general receptor for phosphoinositides 1)-associated scaffold protein	23
1418917_at	heme binding protein 2	3
1415751_at	heterochromatin protein 1, binding protein 3	3
1423051_at	heterogeneous nuclear ribonucleoprotein U	7
1455206_at	hypothetical protein C130006E23	3
1426471_at	hypothetical protein LOC100044274; zinc finger protein 52	3
1423197_a_at	hypothetical protein LOC100044423	23
1440254_at	hypothetical protein LOC100045398	3
1433470_a_at	inner membrane protein, mitochondrial	3
1454755_at	inositol 1,4,5-trisphosphate 3-kinase C	3
1434011_a_at	integrator complex subunit 5	3
1418265_s_at	interferon regulatory factor 2	23
1425280_at	leukocyte receptor cluster (LRC) member 1	23
1421153_at	lysyl oxidase-like 4	7
1429720_at	MAK10 homolog, amino-acid N-acetyltransferase subunit, (S. cerevisiae)	3
1429108_at	male-specific lethal 2 homolog (Drosophila)	7
1434746_at	MAX gene associated	3
1428979_at	metal response element binding transcription factor 1	3

CLUSTER 1 (continued)		
AFFYMETRIX_3PRIME_IVT_ID	Name	Peak phase
1456702_x_at	methionine adenosyltransferase II, alpha	7
1438976_x_at	methionine adenosyltransferase II, alpha	7
1438630_x_at	methionine adenosyltransferase II, alpha	7
1438386_x_at	methionine adenosyltransferase II, alpha	7
1433576_at	methionine adenosyltransferase II, alpha	7
1424219_at	mitochondrial rRNA methyltransferase 1 homolog (S. cerevisiae)	3
1416991_at	mitochondrial translation optimization 1 homolog (S. cerevisiae)	7
1438025_at	mitochondrial translational release factor 1-like	7
1424483_at	MOB1, Mps One Binder kinase activator-like 1B (yeast)	3
1434388_at	MOB1, Mps One Binder kinase activator-like 2A (yeast)	3
1453032_at	MOB1, Mps One Binder kinase activator-like 3 (yeast)	3
1450432_s_at	MUS81 endonuclease homolog (yeast)	7
1451042_a_at	myc induced nuclear antigen	3
1433513_x_at	NADH dehydrogenase (ubiquinone) 1 alpha subcomplex, 12	7
1435737_a_at	nuclear distribution gene E homolog 1 (A nidulans)	11
1458299_s_at	nuclear factor of kappa light polypeptide gene enhancer in B-cells inhibitor, epsilon	3
1428583_at	nuclear fragile X mental retardation protein interacting protein 2	3
1451678_at	nuclear prelamin A recognition factor	3
1419266_at	nuclear transcription factor-Y beta	7
1441733_s_at	nucleoporin 153	3
1429627_at	peptidylprolyl isomerase (cyclophilin)-like 3	3
1428997_at	PHD finger protein 23	3
1451298_at	pleckstrin homology domain containing, family H (with MyTH4 domain) member 3	3
1434139_at	poly (ADP-ribose) polymerase family, member 11	7
1448277_at	polymerase (DNA directed), delta 2, regulatory subunit	3
1435057_x_at	polymerase (RNA) I polypeptide E	3
1435339_at	potassium channel tetramerisation domain containing 15	3
1452625_at	potassium channel tetramerisation domain containing 2	23
1416698_a_at	predicted gene 10124; predicted gene 6340; CDC28 protein kinase 1b	7
1423040_at	predicted gene 11652; predicted gene 5191; basic leucine zipper and W2 domains 1	3
1427965_at	predicted gene 12115; single-stranded DNA binding protein 1	7
1423723_s_at	predicted gene 13886; TAR DNA binding protein	3
1455183_at	predicted gene 3555; predicted gene 7451; serine/threonine kinase 38 like	7
1435878_at	predicted gene 3555; predicted gene 7451; serine/threonine kinase 38 like	3
1416175_a_at	predicted gene 7591; voltage-dependent anion channel 3	3
1428458_at	processing of precursor 1, ribonuclease P/MRP family, (S. cerevisiae)	23
1417591_at	prostaglandin E synthase 2	3
1454236_a_at	protein phosphatase 4, regulatory subunit 1-like; family with sequence similarity 118, member B	3
1423083_at	RAB33B, member of RAS oncogene family	3
1429491_s_at	Rap1 interacting factor 1 homolog (yeast)	7
1428710_at	Ras-like without CAAX 1	3
1417400_at	retinoic acid induced 14	3
1439780_at	ribosomal protein L7-like 1; predicted gene 11222	3
1426386_at	ribosomal protein L7-like 1; predicted gene 11222	3
1455174_at	ribosomal protein S19 binding protein 1	23
1428411_at	RIKEN cDNA 1700020I14 gene	3
1430036_at	RIKEN cDNA 2310015B20 gene	7
1440992_at	RIKEN cDNA 3110052M02 gene	7
1438038_at	RIKEN cDNA 4930402H24 gene	7
1448251_at	RIKEN cDNA 9030425E11 gene	7
1456331_at	RIKEN cDNA 9130023H24 gene	7
1439038_at	RIKEN cDNA 9130227C08Rik gene	7
1444450_at	RIKEN cDNA 9530096D07 gene	23
1424135_at	ring finger and SPRY domain containing 1	23
1423729_a_at	ring finger protein 181	3
1436979_x_at	RNA binding motif protein 14	3
1451321_a_at	RNA binding motif protein 43	3
1424559_at	RNA polymerase II associated protein 2	7
1434131_at	RUN and FYVE domain containing 1	3
1429554_at	serologically defined colon cancer antigen 3	3
1455286_at	similar to BTB (POZ) domain containing 1 (predicted); BTB (POZ) domain containing 1; predicted gene 14121	7
1455643_s_at	similar to CG7338-PA; TSR1, 20S rRNA accumulation, homolog (yeast)	3
1433502_s_at	similar to CG7338-PA; TSR1, 20S rRNA accumulation, homolog (yeast)	3
1426765_at	similar to COMM domain-containing protein 7; COMM domain containing 7; similar to COMM domain containing protein 7	23
1423502_at	similar to mKIAA4005 protein; bromodomain containing 2	7
1434481_at	similar to RIKEN cDNA 4121402D02 gene; male-specific lethal 1 homolog (Drosophila)	23
1451669_at	similar to serine/threonine phosphatase; protein phosphatase 1B, magnesium dependent, beta isoform	3
1438423_at	single-stranded DNA binding protein 2; predicted gene 12470	7
1439399_a_at	small nucleolar RNA host gene (non-protein coding) 1	7

CLUSTER 1 (continued)		
AFFYMETRIX_3PRIME_IVT_ID	Name	Peak phase
1450900_at	SMEK homolog 2, suppressor of mek1 (Dictyostelium)	3
1448385_at	solute carrier family 15, member 4	7
1440569_at	solute carrier family 16 (monocarboxylic acid transporters), member 10	7
1452717_at	solute carrier family 25 (mitochondrial carrier, phosphate carrier), member 24	3
1423796_at	splicing factor proline/glutamine rich (polypyrimidine tract binding protein associated); similar to PTB-associated splicing factor	23
1453722_s_at	splicing factor, arginine/serine-rich 1 (ASF/SF2); similar to splicing factor, arginine/serine-rich 1 (splicing factor 2, alternate splicing factor)	3
1434972_x_at	splicing factor, arginine/serine-rich 1 (ASF/SF2); similar to splicing factor, arginine/serine-rich 1 (splicing factor 2, alternate splicing factor)	3
1428100_at	splicing factor, arginine/serine-rich 1 (ASF/SF2); similar to splicing factor, arginine/serine-rich 1 (splicing factor 2, alternate splicing factor)	23
1424883_s_at	splicing factor, arginine/serine-rich 7	23
1455695_at	ST8 alpha-N-acetyl-neuraminide alpha-2,8-sialyltransferase 1	7
1426771_at	taurine upregulated gene 1	3
1455336_at	THAP domain containing, apoptosis associated protein 2	3
1448994_at	trans-acting transcription factor 1	23
1424610_at	TruB pseudouridine (psi) synthase homolog 2 (E. coli)	7
1460446_at	tryptophan rich basic protein	7
1424644_at	tubulin-specific chaperone C	3
1448147_at	tumor necrosis factor receptor superfamily, member 19	3
1448014_s_at	ubiquitin specific peptidase 24	3
1436635_at	ubiquitin-conjugating enzyme E2Z (putative)	3
1435164_s_at	ubiquitin-like modifier activating enzyme 3	23
1417202_s_at	ubiquitin-like modifier activating enzyme 3	7
1428944_at	ubiquitin-like modifier activating enzyme 6	3
1453162_at	UTP11-like, U3 small nucleolar ribonucleoprotein, (yeast)	7
1424777_at	WD repeat domain 21	7
1434552_at	WD repeat domain 77; predicted gene 15528	3
1422844_a_at	WD repeat domain 77; predicted gene 15528	23
1454922_at	WD repeat domain 92	3
1429125_at	zinc finger and BTB domain containing 9	7
1420095_s_at	zinc finger and SCAN domain containing 21	7
1452374_at	zinc finger protein 322A	23
1439035_at	zinc finger protein 322A	3
1437959_at	zinc finger protein 324	3
1431830_at	zinc finger protein 329	3
1451997_at	zinc finger protein 426	7
1429800_at	zinc finger protein 526	7
1455633_at	zinc finger protein 647	23
1424261_at	zinc finger protein 672	23
1434461_at	zinc finger protein 715	7
1436594_at	zinc finger protein 719	11
1453230_at	zinc finger protein 74; hypothetical protein LOC100044255	7
1440226_at	zinc finger protein 760; hypothetical protein LOC100044275	23
1426293_at	zinc finger protein 790	7
1452319_at	zinc finger protein 82	7
1447947_at	zinc finger, FYVE domain containing 16	3

CLUSTER 2		
AFFYMETRIX_3PRIME_IVT_ID	Name	Peak phase
1428547_at	5' nucleotidase, ecto	19
1421858_at	a disintegrin and metallopeptidase domain 17	23
1424951_at	BAI1-associated protein 2-like 1	23
1429438_at	BCL6 interacting corepressor	3
1434410_at	beta-gamma crystallin domain containing 3	3
1437270_a_at	cardiotrophin-like cytokine factor 1	23
1417132_at	cell division cycle 25 homolog A (S. pombe)	23
1436156_at	cell division cycle and apoptosis regulator 1	23
1419583_at	chromobox homolog 4 (Drosophila Pc class)	23
1435775_at	circadian locomoter output cycles kaput	3
1418659_at	circadian locomoter output cycles kaput	3
1451114_at	CKLF-like MARVEL transmembrane domain containing 6	19
1423792_a_at	CKLF-like MARVEL transmembrane domain containing 6	23
1434953_at	coiled-coil domain containing 93	23
1416814_at	cytotoxic granule-associated RNA binding protein 1	23
1426915_at	death associated protein kinase 1	23
1455711_at	deltex 4 homolog (Drosophila)	3
1460174_at	dexamethasone-induced transcript	23

CLUSTER 2 (continued)		
AFFYMETRIX_3PRIME_IVT_ID	Name	Peak phase
1452908_at	DIP2 disco-interacting protein 2 homolog A (Drosophila); similar to mKIAA0184 protein	3
1436125_at	DNA segment, Chr 16, ERATO Doi 472, expressed	23
1455118_at	DNA segment, Chr 9, ERATO Doi 402, expressed	3
1416755_at	DnaJ (Hsp40) homolog, subfamily B, member 1	3
1418648_at	EGL nine homolog 3 (C. elegans)	3
1435143_at	ELK3, member of ETS oncogene family	3
1451550_at	Eph receptor B3	23
1448510_at	ephrin A1	23
1416895_at	ephrin A1	23
1434105_at	EPM2A (laforin) interacting protein 1	23
1433489_s_at	fibroblast growth factor receptor 2	3
1434344_at	G patch domain and KOW motifs	3
1443997_at	G protein regulated inducer of neurite outgrowth 2	23
1449514_at	G protein-coupled receptor kinase 5	3
1436672_at	G protein-coupled receptor kinase 5	3
1424895_at	G-protein signalling modulator 2 (AGS3-like, C. elegans)	23
1435606_at	galactose-3-O-sulfotransferase 4	23
1434568_at	general transcription factor IIIC, polypeptide 6, alpha	3
1428715_at	glutamine fructose-6-phosphate transaminase 1	23
1436256_at	GrpE-like 2, mitochondrial	23
1435932_at	Hermansky-Pudlak syndrome 6	3
1427144_at	heterogeneous nuclear ribonucleoprotein L-like; glutathione peroxidase 4	23
1455631_at	histocompatibility 13	23
1448704_s_at	histocompatibility 47	7
1425874_at	homeo box C13	23
1418678_at	hyaluronan synthase 2	3
1417981_at	insulin induced gene 2	23
1417980_a_at	insulin induced gene 2	23
1437672_at	insulin receptor substrate 3	23
1423275_at	integrator complex subunit 6	7
1423274_at	integrator complex subunit 6	3
1442350_at	integrin alpha 2; far 3' UTR (from AK028540)	23
1440169_x_at	interferon (alpha and beta) receptor 2	23
1424885_at	kelch domain containing 8A	23
1454637_at	kelch-like 8 (Drosophila)	23
1435472_at	kringle containing transmembrane protein 1	3
1427512_a_at	laminin, alpha 3	19
1435832_at	leucine rich repeat containing 4	3
1416097_at	leucine rich repeat containing 4	3
1454847_at	lipoma HMGIC fusion partner-like 2	7
1416914_s_at	mammary tumor virus receptor 2	3
1448383_at	matrix metallopeptidase 14 (membrane-inserted)	3
1457708_at	methyl-CpG binding domain protein 4	23
1449490_at	methyl-CpG binding domain protein 4	23
1441079_at	methyl-CpG binding domain protein 4	23
1437152_at	mex3 homolog B (C. elegans)	23
1437627_at	mex3 homolog D (C. elegans)	3
1454919_at	N-myristoyltransferase 2	23
1436189_at	NAD(P)H dehydrogenase, quinone 2	23
1421037_at	neuronal PAS domain protein 2	23
1434802_s_at	neurotrophin 3	23
1415926_at	nucleoporin 62	19
1417971_at	nurim (nuclear envelope membrane protein)	23
1455131_at	optic atrophy 3 (human)	23
1428484_at	oxysterol binding protein-like 3	23
1420851_at	par-6 partitioning defective 6 homolog gamma (C. elegans)	3
1416069_at	phosphofructokinase, platelet	3
1435363_at	pleckstrin homology domain containing, family G (with RhoGef domain) member 1	23
1455037_at	plexin A2	3
1451753_at	plexin A2	23
1451475_at	plexin D1	23
1438455_at	poly(A) binding protein, cytoplasmic 4-like	23
1427094_at	polymerase (DNA directed), epsilon 2 (p59 subunit)	3
1430125_s_at	PQ loop repeat containing 1	23
1426567_a_at	PQ loop repeat containing 1	23
1459861_s_at	predicted gene 12397	23
1423911_at	predicted gene 3353; predicted gene 13142; predicted gene 13169; similar to protein phosphatase 2, regulatory subunit B (B56), alpha isoform; predicted gene 13244; predicted gene 13233; protein phosphatase 2, regulatory subunit B (B56), alpha isoform	3
1456846_at	predicted gene 5188	3

CLUSTER 2 (continued)		
AFFYMETRIX_3PRIME_IVT_ID	Name	Peak phase
1423304_a_at	predicted gene 6162; similar to ring finger 111; ring finger 111	3
1448020_at	predicted gene 9392; similar to Raichu404X; RAS-related protein-1a	23
1436993_x_at	profilin 2	3
1452834_at	proline rich 5 like; hypothetical protein LOC100048398	3
1455394_at	protein inhibitor of activated STAT 4	23
1426726_at	protein phosphatase 1, regulatory subunit 10	23
1417479_at	protein phosphatase 2, regulatory subunit B'', gamma; predicted gene 2446; predicted gene 2436	3
1432435_s_at	protein phosphatase 4, regulatory subunit 1-like; family with sequence similarity 118, member B	23
1422541_at	protein tyrosine phosphatase, receptor type, M	3
1416389_a_at	regulator of chromosome condensation (RCC1) and BTB (POZ) domain containing protein 2	3
1416962_at	regulator of chromosome condensation 1	3
1450896_at	Rho GTPase activating protein 5	23
1434422_at	RIKEN cDNA 1700066M21 gene	23
1428337_at	RIKEN cDNA 1810034K20 gene	3
1429061_at	RIKEN cDNA 1810063B05 gene	23
1438972_x_at	RIKEN cDNA 2810410L24 gene	19
1424802_a_at	RIKEN cDNA 3300001P08 gene	3
1433174_a_at	RIKEN cDNA 5430440L12 gene	23
1435928_at	RIKEN cDNA 6430548M08 gene	3
1448250_at	RIKEN cDNA 9030425E11 gene	3
1429987_at	RIKEN cDNA 9930013L23 gene	23
1424134_at	ring finger and SPRY domain containing 1	3
1455412_at	Scm-like with four mbt domains 1	3
1420824_at	sema domain, immunoglobulin domain (Ig), transmembrane domain (TM) and short cytoplasmic domain, (semaphorin) 4D	23
1426248_at	serine/threonine kinase 24 (STE20 homolog, yeast)	23
1421077_at	SERTA domain containing 3	23
1449303_at	sestrin 3	23
1423941_at	similar to Calcium/calmodulin-dependent protein kinase type II gamma chain (CaM-kinase II gamma chain) (CaM kinase II gamma subunit) (CaMK-II subunit gamma); calcium/calmodulin-dependent protein kinase II gamma	23
1450020_at	similar to chemokine receptor CX3CR1; chemokine (C-X3-C) receptor 1	3
1456264_at	similar to COMM domain-containing protein 7; COMM domain containing 7; similar to COMM domain containing protein 7	3
1455106_a_at	similar to creatine kinase, brain; predicted gene 12892; creatine kinase, brain	3
1434790_a_at	similar to Lta4h protein	23
1455703_at	similar to RAC-beta serine/threonine-protein kinase (RAC-PK-beta) (Protein kinase Akt-2) (Protein kinase B, beta) (PKB beta); thymoma viral proto-oncogene 2; similar to serine/threonine kinase	23
1429475_at	similar to RIKEN cDNA 2810457I06	23
1428872_at	similar to RIKEN cDNA 4121402D02 gene; male-specific lethal 1 homolog (Drosophila)	23
1428871_at	similar to RIKEN cDNA 4121402D02 gene; male-specific lethal 1 homolog (Drosophila)	3
1419360_a_at	similar to Ss18 protein; synovial sarcoma translocation, Chromosome 18	23
1418673_at	snail homolog 2 (Drosophila)	23
1421225_a_at	solute carrier family 4 (anion exchanger), member 4	3
1433645_at	solute carrier family 44, member 1	23
1428176_at	sphingosine-1-phosphate receptor 2	3
1417891_at	splA/ryanodine receptor domain and SOCS box containing 3	23
1438458_a_at	splicing factor proline/glutamine rich (polypyrimidine tract binding protein associated); similar to PTB-associated splicing factor	3
1423795_at	splicing factor proline/glutamine rich (polypyrimidine tract binding protein associated); similar to PTB-associated splicing factor	3
1457265_at	splicing factor, arginine/serine-rich 17b	23
1458175_at	spondin 2, extracellular matrix protein	3
1417860_a_at	spondin 2, extracellular matrix protein	3
1433575_at	SRY-box containing gene 19; SRY-box containing gene 4	23
1418546_a_at	STAM binding protein like 1	19
1452276_at	SWI/SNF-related, matrix-associated actin-dependent regulator of chromatin, subfamily a, containing DEAD/H box 1	3
1433909_at	synaptotagmin XVII	3
1445667_at	TBC1 domain family, member 10a	3
1450662_at	testis specific protein kinase 1	3
1424568_at	tetraspanin 2	19
1424567_at	tetraspanin 2	3
1443952_at	thyroid hormone receptor alpha; similar to thyroid hormone receptor	3
1426997_at	thyroid hormone receptor alpha; similar to thyroid hormone receptor	23
1457676_at	toll-interleukin 1 receptor (TIR) domain-containing adaptor protein	3
1436460_at	transmembrane protein 194	3
1437910_at	transmembrane protein 39b	23
1448551_a_at	tripartite motif-containing 2	23
1417028_a_at	tripartite motif-containing 2	23
1417027_at	tripartite motif-containing 2	23

CLUSTER 2 (continued)		
AFFYMETRIX_3PRIME_IVT_ID	Name	Peak phase
1448101_s_at	tripartite motif-containing 27	3
1438376_s_at	tripartite motif-containing 27	3
1451663_a_at	tripartite motif-containing 3	3
1439440_x_at	twinfilin, actin-binding protein, homolog 2 (Drosophila)	23
1418733_at	twist homolog 1 (Drosophila)	23
1436805_at	ubiquitin associated and SH3 domain containing, B	23
1439302_at	ubiquitin-like modifier activating enzyme 6	3
1460688_s_at	unc-119 homolog B (C. elegans)	23
1427933_at	vacuolar protein sorting 33B (yeast)	23
1452898_at	vacuolar protein sorting 36 (yeast)	7
1434708_at	von Hippel-Lindau syndrome	3
1451649_a_at	WD repeat domain 75	3
1428616_at	zinc finger protein 131; similar to putative transcription factor ZNF131	19
1420944_at	zinc finger protein 185	3
1420943_at	zinc finger protein 185	3
1434689_at	zinc finger protein 637	3
1433748_at	zinc finger, DHHC domain containing 18	23
1428052_a_at	zinc finger, MYM domain containing 1	3
1452970_at	zinc finger, MYM-type 2	23

CLUSTER 3		
AFFYMETRIX_3PRIME_IVT_ID	Name	Peak phase
1428821_at	1-acylglycerol-3-phosphate O-acyltransferase 2 (lysophosphatidic acid acyltransferase, beta)	7
1448318_at	adipose differentiation related protein	7
1450706_a_at	ADP-ribosylation factor-like 3	7
1457021_x_at	anti-Mullerian hormone type 2 receptor	7
1439021_at	ArfGAP with coiled-coil, ankyrin repeat and PH domains 3	3
1423122_at	arginine vasopressin-induced 1	7
1434543_a_at	bolA-like 2 (E. coli)	7
1416257_at	calpain 2	7
1452901_at	cAMP responsive element binding protein 1	7
1448347_a_at	cell cycle associated protein 1	7
1436034_at	centrosomal protein 68	7
1422636_at	cyclin D binding myb-like transcription factor 1	7
1434435_s_at	cytochrome c oxidase, subunit XVII assembly protein homolog (yeast)	7
1450654_a_at	dehydrodolichyl diphosphate synthase	7
1420368_at	density-regulated protein	3
1420129_s_at	DNA segment, Chr 10, Wayne State University 52, expressed	7
1448900_at	DNA segment, Chr 16, human D22S680E, expressed	7
1435117_a_at	DnaJ (Hsp40) homolog, subfamily C, member 27	3
1452594_at	dual specificity phosphatase 11 (RNA/RNP complex 1-interacting)	7
1452153_at	F-box protein 18	7
1447904_s_at	farnesyltransferase, CAAX box, alpha; similar to farnesyltransferase alpha subunit	7
1426369_at	fatty acyl CoA reductase 1	3
1435315_s_at	fatty acyl CoA reductase 1	11
1438702_at	fibronectin leucine rich transmembrane protein 2	7
1416859_at	FK506 binding protein 3	11
1417113_at	germ cell-less homolog 1 (Drosophila)	7
1448649_at	glutamyl aminopeptidase	7
1451467_s_at	GTP binding protein 5	7
1438180_x_at	HCLS1 associated X-1; silica-induced gene 111	7
1435194_at	heat shock protein 4	11
1415872_at	heterogeneous nuclear ribonucleoprotein H1	7
1423050_s_at	heterogeneous nuclear ribonucleoprotein U	7
1434551_at	heterogeneous nuclear ribonucleoprotein U-like 2	7
1424017_a_at	histidine triad nucleotide binding protein 1	7
1417024_at	histidyl-tRNA synthetase	7
1436172_at	hypothetical protein 9530028C05	7
1418116_at	interferon alpha responsive gene	7
1424746_at	kinesin family member 1C	7
1425991_a_at	KN motif and ankyrin repeat domains 2	3
1425141_at	lactamase, beta 2	7
1429071_at	malic enzyme 3, NADP(+)-dependent, mitochondrial	7
1416424_at	mannose-6-phosphate receptor binding protein 1	7
1417234_at	matrix metallopeptidase 11	7
1416759_at	microtubule associated monoxygenase, calponin and LIM domain containing 1	7
1448190_at	mitochondrial ribosomal protein L33	7
1460701_a_at	mitochondrial ribosomal protein L52	7
1434815_a_at	mitogen-activated protein kinase-activated protein kinase 3	7

CLUSTER 3 (continued)		
AFFYMETRIX_3PRIME_IVT_ID	Name	Peak phase
1448979_at	muted	7
1426052_at	mutL homolog 3 (E coli)	7
1459991_at	myosin IXa	7
1426464_at	nuclear receptor subfamily 1, group D, member 1	7
1448476_at	nucleosome assembly protein 1-like 4	7
1431302_a_at	nudix (nucleoside diphosphate linked moiety X)-type motif 7	3
1428309_s_at	p53 and DNA damage regulated 1	3
1448432_at	phospholipase C, delta 1	7
1417138_s_at	polymerase (RNA) II (DNA directed) polypeptide E	11
1434383_at	praja 2, RING-H2 motif containing	7
1452083_a_at	praja1, RING-H2 motif containing	3
1438940_x_at	predicted gene 11663; predicted gene 2992; predicted gene 7862; high mobility group nucleosomal binding domain 1; similar to high mobility group nucleosomal binding domain 1	7
1427174_at	predicted gene 2004	7
1444589_at	predicted gene 4944	7
1439438_a_at	predicted gene 6563; SAP domain containing ribonucleoprotein	7
1417998_at	predicted gene 9769; prostaglandin E synthase 3 (cytosolic); similar to Sid3177p; predicted gene 11893	7
1436846_x_at	predicted gene, EG546165; predicted gene 2423; hypothetical protein LOC674211; tyrosine 3-monooxygenase/tryptophan 5-monooxygenase activation protein, theta polypeptide	7
1460590_s_at	predicted gene, EG546165; predicted gene 2423; hypothetical protein LOC674211; tyrosine 3-monooxygenase/tryptophan 5-monooxygenase activation protein, theta polypeptide	7
1460621_x_at	predicted gene, EG546165; predicted gene 2423; hypothetical protein LOC674211; tyrosine 3-monooxygenase/tryptophan 5-monooxygenase activation protein, theta polypeptide	7
1448528_at	programmed cell death 10	7
1418840_at	programmed cell death 4	7
1460198_a_at	proteasome (prosome, macropain) subunit, beta type 3	7
1437234_x_at	protein arginine N-methyltransferase 2	7
1416400_at	pyrroline-5-carboxylate reductase-like	7
1418924_at	Ras association (RalGDS/AF-6) domain family (N-terminal) member 7	7
1460543_x_at	ribosomal protein L37a; predicted gene 4149; predicted gene 4613; predicted gene 14251; predicted gene 14173	7
1435546_a_at	RIKEN cDNA 1810013L24 gene	7
1452043_at	RIKEN cDNA 2310011J03 gene	7
1428083_at	RIKEN cDNA 2310043N10 gene	7
1416577_a_at	ring-box 1; predicted gene 9840	7
1454815_at	SERTA domain containing 2	11
1448316_at	similar to CKLF-like MARVEL transmembrane domain containing 3	11
1456125_a_at	similar to cytoplasmic dynein light chain 1; predicted gene 11582; dynein light chain LC8-type 1; predicted gene 6788	3
1456898_at	similar to hCG45299; purine rich element binding protein A	7
1454627_a_at	similar to ribosomal protein; predicted gene 6344; predicted gene 11449; predicted gene 13841; predicted gene 8210; hypothetical protein LOC675793; ribosomal protein L29; predicted gene 12704; predicted gene 7252; predicted gene 8965; predicted gene 13213; predicted gene 8580; predicted gene 15526; predicted gene 7934; predicted gene 5218; predicted gene 5561; predicted gene 8548; predicted gene 12447; predicted gene 8852; predicted gene 12508; predicted gene 6010; predicted gene 13921; predicted gene 8860; predicted gene 13545; predicted gene 9405; predicted gene 6862; predicted gene 13141; predicted gene 10709	7
1449815_a_at	single-stranded DNA binding protein 2; predicted gene 12470	11
1417648_s_at	sorting nexin 5	7
1452439_s_at	splicing factor, arginine/serine-rich 2 (SC-35)	7
1449079_s_at	ST3 beta-galactoside alpha-2,3-sialyltransferase 6	3
1423350_at	suppressor of cytokine signaling 5	7
1435982_at	syntaxin 12	7
1418164_at	syntaxin 2	7
1448028_at	TBC1 domain family, member 24	7
1435334_at	tetratricopeptide repeat domain 7	7
1424383_at	transmembrane protein 51	7
1454797_at	transmembrane protein 55b	3
1428747_at	tRNA selenocysteine 1 associated protein 1	7
1420880_a_at	tyrosine 3-monooxygenase/tryptophan 5-monooxygenase activation protein, beta polypeptide	7
1423138_at	WD repeat domain 4	7
1428629_at	zinc finger protein 518	7
1424670_s_at	zinc finger, FYVE domain containing 21	7

CLUSTER 4		
AFFYMETRIX_3PRIME_IVT_ID	Name	Peak phase
1434087_at	5,10-methylenetetrahydrofolate reductase	23
1452595_at	a disintegrin-like and metallopeptidase (reprolysin type) with thrombospondin type 1 motif, 4	23
1455965_at	a disintegrin-like and metallopeptidase (reprolysin type) with thrombospondin type 1 motif, 4	23
1425099_a_at	aryl hydrocarbon receptor nuclear translocator-like	19
1428982_at	ATPase family, AAA domain containing 2B	19

CLUSTER 4 (continued)		
AFFYMETRIX_3PRIME_IVT_ID	Name	Peak phase
1447139_at	B-cell CLL/lymphoma 7C	23
1436960_at	bromodomain containing 3	23
1420907_at	CD2-associated protein	23
1434957_at	cell adhesion molecule-related/down-regulated by oncogenes	23
1418660_at	circadian locomoter output cycles kaput	19
1438024_at	coiled-coil domain containing 90A	23
1434326_x_at	coronin, actin binding protein, 2B	19
1434755_at	coronin, actin binding protein, 2B	23
1436914_at	CTD (carboxy-terminal domain, RNA polymerase II, polypeptide A) small phosphatase like 2	19
1420617_at	cytoplasmic polyadenylation element binding protein 4	19
1423042_at	DEAD/H (Asp-Glu-Ala-Asp/His) box polypeptide 3, X-linked	19
1451453_at	death-associated protein kinase 2	19
1419204_at	delta-like 1 (Drosophila)	23
1419995_at	DNA segment, Chr 10, ERATO Doi 641, expressed	23
1439332_at	DNA-damage-inducible transcript 4-like	23
1444139_at	DNA-damage-inducible transcript 4-like	23
1451751_at	DNA-damage-inducible transcript 4-like	19
1420722_at	elongation of very long chain fatty acids (FEN1/Elo2, SUR4/Elo3, yeast)-like 3	3
1427069_at	F-box protein 28	23
1437150_at	family with sequence similarity 110, member B	23
1440359_at	family with sequence similarity 110, member B	23
1452924_at	family with sequence similarity 83, member D	23
1460251_at	Fas (TNF receptor superfamily member 6)	19
1434699_at	G2/M-phase specific E3 ubiquitin ligase	23
1419359_at	hexamethylene bis-acetamide inducible 1	19
1418415_at	homeo box B5	23
1438274_at	IKAROS family zinc finger 4; similar to helios	23
1417612_at	immediate early response 5	23
1422053_at	inhibin beta-A	19
1435465_at	kelch repeat and BTB (POZ) domain containing 11	23
1424594_at	lectin, galactose binding, soluble 7	3
1436356_at	lectin, galactose binding, soluble 7	19
1455293_at	Leo1, Paf1/RNA polymerase II complex component, homolog (S. cerevisiae)	23
1434424_at	major facilitator superfamily domain containing 7B	3
1424180_a_at	mediator complex subunit 24	23
1437404_at	microtubule associated serine/threonine kinase family member 4	23
1416733_at	muskelin 1, intracellular mediator containing kelch motifs	23
1426717_at	non imprinted in Prader-Willi/Angelman syndrome 2 homolog (human)	23
1447905_x_at	nucleoporin 62	19
1459441_at	odd Oz/ten-m homolog 3 (Drosophila)	3
1418302_at	palmitoyl-protein thioesterase 2	23
1435562_at	PDZ domain containing 8	23
1416846_a_at	PDZ domain containing RING finger 3	19
1435066_at	phosphatidylinositol transfer protein, cytoplasmic 1	23
1455204_at	phosphatidylinositol transfer protein, cytoplasmic 1	23
1415806_at	plasminogen activator, tissue	19
1455271_at	predicted gene 13889	23
1428615_at	purinergic receptor P2Y, G-protein coupled, 5	19
1418892_at	ras homolog gene family, member J	19
1428227_at	RE1-silencing transcription factor	23
1448689_at	related RAS viral (r-ras) oncogene homolog 2	19
1424163_at	required for meiotic nuclear division 5 homolog B (S. cerevisiae)	3
1427522_at	Rho GTPase activating protein 20	23
1429918_at	Rho GTPase activating protein 20	19
1454745_at	Rho GTPase activating protein 29	19
1424318_at	RIKEN cDNA 1110067D22 gene	23
1440062_at	RIKEN cDNA 2510009E07 gene	23
1429451_at	RIKEN cDNA 2610301B20 gene	23
1442307_at	RIKEN cDNA B130063F10 gene	23
1448005_at	SAM and SH3 domain containing 1; predicted gene 2082	23
1445957_at	similar to Ena-VASP-like; Ena-vasodilator stimulated phosphoprotein	19
1434521_at	similar to regulatory factor X domain containing 2 homolog; regulatory factor X, 7	23
1415802_at	solute carrier family 16 (monocarboxylic acid transporters), member 1	3
1434096_at	solute carrier family 4 (anion exchanger), member 4	23
1452071_at	solute carrier family 4 (anion exchanger), member 4	23
1438673_at	solute carrier family 4, sodium bicarbonate cotransporter, member 7	19
1417061_at	solute carrier family 40 (iron-regulated transporter), member 1	23
1457686_at	splicing factor, arginine/serine-rich 17b	23
1436584_at	sprouty homolog 2 (Drosophila)	19
1419155_a_at	SRY-box containing gene 19; SRY-box containing gene 4	23

CLUSTER 4 (continued)		
AFFYMETRIX_3PRIME_IVT_ID	Name	Peak phase
1419156_at	SRY-box containing gene 19; SRY-box containing gene 4	23
1449370_at	SRY-box containing gene 19; SRY-box containing gene 4	23
1428831_at	SUMO/sentrin specific peptidase 5	3
1421594_a_at	synaptotagmin-like 2	23
1421529_a_at	thioredoxin reductase 1	23
1419132_at	toll-like receptor 2	23
1456009_x_at	topoisomerase (DNA) III beta	23
1428108_x_at	transmembrane and coiled-coil domains 2	23
1454730_at	transmembrane anterior posterior transformation 1	19
1433735_a_at	transmembrane protein 64	23
1428281_at	TruB pseudouridine (psi) synthase homolog 1 (E. coli)	19
1449033_at	tumor necrosis factor receptor superfamily, member 11b (osteoprotegerin)	23
1418571_at	tumor necrosis factor receptor superfamily, member 12a	3
1418572_x_at	tumor necrosis factor receptor superfamily, member 12a	3
1442590_at	tumor necrosis factor receptor superfamily, member 23	23
1436308_at	zinc finger protein 292	23

CLUSTER 5		
AFFYMETRIX_3PRIME_IVT_ID	Name	Peak phase
1443968_at	adenosine deaminase, RNA-specific, B1	15
1423420_at	adrenergic receptor, beta 1	11
1416077_at	adrenomedullin	11
1447839_x_at	adrenomedullin	11
1418901_at	CCAAT/enhancer binding protein (C/EBP), beta	15
1427844_a_at	CCAAT/enhancer binding protein (C/EBP), beta	15
1458040_at	cerberus 1 homolog (Xenopus laevis)	15
1450488_at	chemokine (C-C motif) ligand 24	11
1416953_at	connective tissue growth factor	15
1416537_at	cysteine-rich with EGF-like domains 1	15
1437763_at	DCN1, defective in cullin neddylation 1, domain containing 3 (S. cerevisiae)	15
1447462_at	DNA segment, Chr 7, Wayne State University 130, expressed	15
1431422_a_at	dual specificity phosphatase 14	15
1434944_at	dystrophia myotonica-protein kinase	11
1437829_s_at	eukaryotic elongation factor-2 kinase	15
1439860_at	eukaryotic elongation factor-2 kinase	15
1449013_at	eukaryotic elongation factor-2 kinase	11
1434414_at	FAD-dependent oxidoreductase domain containing 2	11
1451099_at	family with sequence similarity 62 (C2 domain containing), member A	15
1441042_at	fibroblast growth factor 1	15
1416125_at	FK506 binding protein 5	15
1448231_at	FK506 binding protein 5	11
1417429_at	flavin containing monooxygenase 1	11
1437408_at	G protein-coupled receptor 126	15
1418483_a_at	glycoprotein galactosyltransferase alpha 1, 3	11
1451843_a_at	glycoprotein galactosyltransferase alpha 1, 3	15
1434735_at	hepatic leukemia factor	11
1434736_at	hepatic leukemia factor	11
1428584_a_at	hydroxyacylglutathione hydrolase-like	7
1460675_at	immunoglobulin superfamily, member 8	19
1436987_at	isthmin 1 homolog (zebrafish)	15
1436988_at	isthmin 1 homolog (zebrafish)	15
1444165_at	layilin	11
1421101_a_at	LIM domain binding 2	11
1439557_s_at	LIM domain binding 2	11
1455665_at	LON peptidase N-terminal domain and ring finger 1	11
1436830_at	MARVEL (membrane-associating) domain containing 1	15
1449901_a_at	mitogen-activated protein kinase kinase kinase 6	15
1417663_a_at	N-myc downstream regulated gene 3	11
1428393_at	neuritin 1	11
1433907_at	Pbx/knotted 1 homeobox 2	11
1449851_at	period homolog 1 (Drosophila)	15
1417602_at	period homolog 2 (Drosophila)	15
1457350_at	period homolog 2 (Drosophila)	11
1421087_at	period homolog 3 (Drosophila)	15
1455345_at	PHD finger protein 15	15
1430966_at	predicted gene 4477; camello-like 3	15
1425328_at	predicted gene 7527; family with sequence similarity 76, member A	19
1460674_at	progestin and adipoQ receptor family member VII	11
1451576_at	protein kinase, DNA activated, catalytic polypeptide	15

CLUSTER 5 (continued)		
AFFYMETRIX_3PRIME_IVT_ID	Name	Peak phase
1427929_a_at	pyridoxal (pyridoxine, vitamin B6) kinase	11
1442067_at	receptor tyrosine kinase-like orphan receptor 1	15
1451171_at	RIKEN cDNA 2310008H04 gene	15
1457438_at	RIKEN cDNA 2310030N02 gene	11
1436673_at	RIKEN cDNA 2610020H08 gene	15
1441799_at	RIKEN cDNA 6030422H21 gene	15
1436316_at	RIKEN cDNA 9430029L20 gene	15
1426792_s_at	RUN and SH3 domain containing 2	7
1419766_at	salt inducible kinase 1	15
1420588_at	serine protease inhibitor-like, with Kunitz and WAP domains 1 (eppin)	11
1439802_at	serine/threonine kinase 35	15
1456640_at	SH3 domain containing ring finger 2	15
1455794_at	smoothelin-like 2	11
1420150_at	splA/ryanodine receptor domain and SOCS box containing 1	15
1428472_at	splA/ryanodine receptor domain and SOCS box containing 1	15
1449752_at	splA/ryanodine receptor domain and SOCS box containing 1	11
1434963_at	suppressor of Ty 3 homolog (S. cerevisiae)	11
1425484_at	thymocyte selection-associated high mobility group box; similar to thymus high mobility group box protein TOX	11
1442039_at	thymocyte selection-associated high mobility group box; similar to thymus high mobility group box protein TOX	11
1455109_at	transducin (beta)-like 1X-linked receptor 1	15
1418158_at	transformation related protein 63	15
1420772_a_at	TSC22 domain family, member 3	11
1425281_a_at	TSC22 domain family, member 3	11
1433699_at	tumor necrosis factor, alpha-induced protein 3	15
1428731_at	ubiquitin specific peptidase 54	15
1416773_at	WEE 1 homolog 1 (S. pombe)	15
1416774_at	WEE 1 homolog 1 (S. pombe)	11
1419874_x_at	zinc finger and BTB domain containing 16	15
1439163_at	zinc finger and BTB domain containing 16	15
1442025_a_at	zinc finger and BTB domain containing 16	15
1442026_at	zinc finger and BTB domain containing 16	15
1459557_at	zinc finger and BTB domain containing 16	7

CLUSTER 6		
AFFYMETRIX_3PRIME_IVT_ID	Name	Peak phase
1456901_at	a disintegrin-like and metallopeptidase (reprolysin type) with thrombospondin type 1 motif, 20	19
1418382_at	adenomatosis polyposis coli down-regulated 1	19
1418383_at	adenomatosis polyposis coli down-regulated 1	19
1437604_x_at	adenomatosis polyposis coli down-regulated 1	15
1449070_x_at	adenomatosis polyposis coli down-regulated 1	19
1434932_at	adenosine deaminase, RNA-specific, B1	15
1460020_at	ankyrin repeat domain 11	19
1424712_at	AT hook containing transcription factor 1	19
1452929_at	CAP-GLY domain containing linker protein 1	15
1434565_at	cell growth regulator with ring finger domain 1	19
1433733_a_at	cryptochrome 1 (photolyase-like)	23
1435975_at	DENN/MADD domain containing 4A	19
1453027_at	discs, large (Drosophila) homolog-associated protein 1	15
1419353_at	dolichol-phosphate (beta-D) mannosyltransferase 1	15
1428222_at	doublecortin-like kinase 2	15
1429250_at	dynein cytoplasmic 2 heavy chain 1	19
1417235_at	EH-domain containing 3	19
1435719_at	expressed sequence AI448984	15
1435280_at	expressed sequence AI452195	15
1459504_at	expressed sequence AU022121	15
1457442_at	expressed sequence AW125324	15
1445049_at	expressed sequence C80893	15
1436580_at	heparan-alpha-glucosaminide N-acetyltransferase	15
1428373_at	hypothetical protein LOC100044300; inositol hexaphosphate kinase 2	19
1419128_at	integrin alpha X	19
1424259_at	lipase maturation factor 1	19
1434690_at	lysocardiolipin acyltransferase 1	15
1452188_at	mastermind like 1 (Drosophila)	19
1434698_at	melanoma inhibitory activity 3	15
1436502_at	mitochondrial tumor suppressor 1	19
1440609_at	mitogen-activated protein kinase kinase kinase kinase 4	19
1450511_at	muscle, skeletal, receptor tyrosine kinase	15
1429049_at	NUAK family, SNF1-like kinase, 2	15
1435771_at	phospholipase C, beta 4	19

CLUSTER 6 (continued)		
AFFYMETRIX_3PRIME_IVT_ID	Name	Peak phase
1458553_at	plasminogen activator, tissue	15
1456066_a_at	polymerase (RNA) I polypeptide A	15
1421760_at	pre T-cell antigen receptor alpha	15
1449607_at	predicted gene 4210	15
1420304_x_at	predicted gene 4833; predicted gene 9616; predicted gene 13680; predicted gene 4859; predicted gene 8729; predicted gene 8231; ribosomal protein S4, X-linked	15
1451264_at	predicted gene 5780; FERM domain containing 6	19
1430735_at	protein associated with topoisomerase II homolog 2 (yeast)	19
1424886_at	protein tyrosine phosphatase, receptor type, D	15
1435537_at	protein tyrosine phosphatase, receptor type, D	15
1451253_at	PX domain containing serine/threonine kinase; similar to PX domain-containing protein kinase-like protein (Modulator of Na,K-ATPase) (MONaKA)	19
1434062_at	RAB GTPase activating protein 1-like	19
1452833_at	Rap guanine nucleotide exchange factor (GEF) 2	15
1424034_at	RAR-related orphan receptor alpha	15
1458145_at	RAR-related orphan receptor alpha	19
1436059_at	regulatory factor X, 1 (influences HLA class II expression)	19
1433161_at	RIKEN cDNA 1700008N17 gene	15
1437654_at	RIKEN cDNA 3110001K24 gene	19
1453976_at	RIKEN cDNA 4432414F05 gene	19
1441338_at	RIKEN cDNA 5930412G12 gene	15
1458371_at	RIKEN cDNA E130006N16 gene	19
1449944_a_at	Sec61, alpha subunit 2 (S. cerevisiae)	15
1416168_at	serine (or cysteine) peptidase inhibitor, clade F, member 1	15
1455437_at	similar to Serine/threonine-protein kinase QSK; cDNA sequence BC033915	19
1420762_a_at	similar to Y box protein 2; Y box protein 2	15
1426109_a_at	solute carrier family 14 (urea transporter), member 2	15
1426595_at	solute carrier family 18 (vesicular monoamine), member 1	19
1426069_s_at	solute carrier family 7 (cationic amino acid transporter, y+ system), member 4	19
1437924_at	Son DNA binding protein	19
1422822_at	StAR-related lipid transfer (START) domain containing 5	19
1450769_s_at	StAR-related lipid transfer (START) domain containing 5	19
1455171_at	suppressor of variegation 4-20 homolog 1 (Drosophila)	19
1433778_at	tankyrase, TRF1-interacting ankyrin-related ADP-ribose polymerase	15
1435350_at	TNF receptor-associated factor 6	19
1443288_at	TNF receptor-associated factor 6	19
1433795_at	transforming growth factor, beta receptor III	15
1456583_x_at	transmembrane protein 144	15
1445693_at	v-raf murine sarcoma 3611 viral oncogene homolog	19
1426776_at	Wiskott-Aldrich syndrome-like (human)	19
1437023_at	zinc finger protein 12	19
1457552_at	zinc finger protein 295	19
1419787_a_at	zinc finger protein 628	15
1460441_at	zinc finger, X-linked, duplicated B	19

CLUSTER 7		
AFFYMETRIX_3PRIME_IVT_ID	Name	Peak phase
1433477_at	active BCR-related gene	23
1435880_at	ankyrin repeat domain 50	19
1452632_at	AP2 associated kinase 1	19
1415813_at	apoptosis inhibitor 5	19
1434506_at	AT rich interactive domain 2 (ARID, RFX-like); RIKEN cDNA 1700124K17 gene	19
1424702_a_at	ATG2 autophagy related 2 homolog B (S. cerevisiae)	19
1435501_at	ATG2 autophagy related 2 homolog B (S. cerevisiae)	15
1436174_at	ATPase family, AAA domain containing 2	23
1426083_a_at	B-cell translocation gene 1, anti-proliferative; similar to myocardial vascular inhibition factor	19
1448953_at	Bloom syndrome homolog (human)	23
1458755_at	cell adhesion molecule-related/down-regulated by oncogenes	19
1434270_at	chromobox homolog 6; neuronal pentraxin receptor; Cbx6-Nptxr readthrough transcripts	15
1456280_at	claspin homolog (Xenopus laevis)	19
1419430_at	cytochrome P450, family 26, subfamily a, polypeptide 1	19
1420618_at	cytoplasmic polyadenylation element binding protein 4	23
1419270_a_at	deoxyuridine triphosphatase	19
1428299_at	dual-specificity tyrosine-(Y)-phosphorylation regulated kinase 1a; dual-specificity tyrosine-(Y)-phosphorylation regulated kinase 1c	19
1417730_at	exostoses (multiple) 1	19
1454002_at	F-box and WD-40 domain protein 2; possible far 3' UTR of Fbxw2	19
1434002_at	forkhead box N3	19
1434086_at	G protein-coupled receptor 107; hypothetical protein LOC100047589	23

CLUSTER 7 (continued)		
AFFYMETRIX_3PRIME_IVT_ID	Name	Peak phase
1437474_at	GATA zinc finger domain containing 2B	23
1440238_at	glioma tumor suppressor candidate region gene 1	23
1421436_at	glutamate receptor, ionotropic, delta 2	23
1448926_at	homeo box A5	19
1444010_at	hypothetical LOC630527; eukaryotic translation initiation factor 4E; similar to eukaryotic translation initiation factor 4E	19
1433891_at	leucine-rich repeat-containing G protein-coupled receptor 4	19
1450207_at	leukemia inhibitory factor receptor	19
1460249_at	ligand of numb-protein X 2	19
1429863_at	LON peptidase N-terminal domain and ring finger 3	19
1426769_s_at	mastermind like 1 (Drosophila)	19
1434562_at	microfibrillar-associated protein 3	23
1455117_at	minichromosome maintenance complex component 9	19
1423699_at	non-SMC condensin II complex, subunit H2	19
1443846_x_at	pantothenate kinase 2 (Hallervorden-Spatz syndrome)	19
1426994_at	PH domain and leucine rich repeat protein phosphatase 1	19
1418835_at	pleckstrin homology-like domain, family A, member 1	19
1450443_at	polypyrimidine tract binding protein 1; predicted gene 4900	19
1437419_at	predicted gene 4521; BMP2 inducible kinase	19
1425333_at	RAB43, member RAS oncogene family	19
1433900_at	RIKEN cDNA 0610007L01 gene	19
1459967_at	RIKEN cDNA 1810063B05 gene	19
1428561_at	RIKEN cDNA 2610002J23 gene	19
1440849_at	RIKEN cDNA 6330417G04 gene	19
1455680_at	RIKEN cDNA 9630025H16 gene	19
1455554_at	RIKEN cDNA A830039N20 gene	15
1425282_at	ring finger protein 144B	19
1424588_at	SLIT-ROBO Rho GTPase activating protein 3	23
1438187_at	solute carrier family 25 (mitochondrial carrier, palmitoylcarnitine transporter), member 29	15
1438188_x_at	solute carrier family 25 (mitochondrial carrier, palmitoylcarnitine transporter), member 29	15
1433595_at	solute carrier family 35 (UDP-glucuronic acid/UDP-N-acetylgalactosamine dual transporter), member D1; similar to solute carrier family 35 (UDP-glucuronic acid/UDP-N-acetylgalactosamine dual transporter), member D1	19
1434104_at	solute carrier family 35, member E1	19
1416359_at	sorting nexin 18	19
1429029_at	sphingomyelin synthase 2	19
1434966_at	splicing factor, arginine/serine-rich 8	23
1422571_at	thrombospondin 2	23
1449388_at	thrombospondin 4	19
1435344_at	transcription factor Dp 2	19
1421096_at	transient receptor potential cation channel, subfamily C, member 1	19
1434500_at	tweety homolog 2 (Drosophila); similar to tweety homolog 2	19
1434901_at	zinc finger and BTB domain containing 2; predicted gene 13294	23
1437111_at	zinc finger CCCH type containing 12C	19
1424892_at	zinc finger with KRAB and SCAN domains 5	19

CLUSTER 8		
AFFYMETRIX_3PRIME_IVT_ID	Name	Peak phase
1416137_at	annexin A7	11
1416138_at	annexin A7	11
1418025_at	basic helix-loop-helix family, member e40	11
1417161_at	CDK2-associated protein 2	11
1436195_at	cDNA sequence BC046404; similar to CDNA sequence BC046404	7
1427030_at	coiled-coil domain containing 52	7
1455821_x_at	complement component 1, q subcomponent binding protein	11
1418174_at	D site albumin promoter binding protein	7
1438211_s_at	D site albumin promoter binding protein	7
1420512_at	dickkopf homolog 2 (Xenopus laevis)	7
1415691_at	discs, large homolog 1 (Drosophila); similar to Discs, large homolog 1 (Drosophila)	11
1419978_s_at	DNA segment, Chr 10, ERATO Doi 610, expressed	7
1427043_s_at	ecto-NOX disulfide-thiol exchanger 2	11
1424157_at	EH-domain containing 2	7
1448491_at	enoyl coenzyme A hydratase 1, peroxisomal	11
1435547_at	ENSMUSG00000075401	7
1454021_a_at	exosome component 10	15
1455428_at	family with sequence similarity 53, member B	7
1450694_at	FK506 binding protein 2	11
1448559_at	flotillin 1	7
1417220_at	fumarylacetoacetate hydrolase	7
1428767_at	gasdermin D	7

CLUSTER 8 (continued)		
AFFYMETRIX_3PRIME_IVT_ID	Name	Peak phase
1428630_x_at	hydroxyacylglutathione hydrolase-like	11
1417886_at	hypothetical protein LOC100048454; RIKEN cDNA 1810009A15 gene	11
1428487_s_at	hypothetical protein LOC675736; coenzyme Q10 homolog B (S. cerevisiae); predicted gene 4899	11
1418125_at	INO80 homolog (S. cerevisiae); similar to yeast INO80-like protein	7
1449942_a_at	integrin linked kinase; predicted gene 6263	7
1451016_at	interferon-related developmental regulator 2	11
1434541_x_at	KH domain containing, RNA binding, signal transduction associated 1	7
1437389_x_at	KH domain containing, RNA binding, signal transduction associated 1	7
1438462_x_at	KH domain containing, RNA binding, signal transduction associated 1	7
1460559_at	KN motif and ankyrin repeat domains 2	7
1448839_at	KN motif and ankyrin repeat domains 3	11
1417638_at	left right determination factor 1	11
1417592_at	mechanistic target of rapamycin (serine/threonine kinase)	7
1440209_at	membrane-associated ring finger (C3HC4) 1	11
1448486_at	methylmalonyl-Coenzyme A mutase	11
1428709_a_at	mitochondrial ribosomal protein L24	11
1451555_at	neurolysin (metallopeptidase M3 family)	7
1454748_at	nicotinate phosphoribosyltransferase domain containing 1	7
1416958_at	nuclear receptor subfamily 1, group D, member 2; predicted gene 5827	11
1457250_x_at	nuclear receptor subfamily 1, group D, member 2; predicted gene 5827	11
1451974_at	oxysterol binding protein-like 2	7
1441445_at	period homolog 3 (Drosophila)	7
1442243_at	period homolog 3 (Drosophila)	7
1458176_at	period homolog 3 (Drosophila)	7
1454832_at	phosphatase and actin regulator 1	11
1456606_a_at	phosphatase and actin regulator 1	11
1435945_a_at	potassium intermediate/small conductance calcium-activated channel, subfamily N, member 4	7
1435188_at	predicted gene 129	11
1437449_at	radical S-adenosyl methionine domain containing 1	7
1433867_at	RIKEN cDNA 1810030O07 gene	7
1457826_a_at	RIKEN cDNA 1810043H04 gene	7
1418245_a_at	RNA binding motif protein 9	7
1419149_at	serine (or cysteine) peptidase inhibitor, clade E, member 1	11
1423851_a_at	shisa homolog 2 (Xenopus laevis)	11
1423852_at	shisa homolog 2 (Xenopus laevis)	11
1419649_s_at	similar to nuclear myosin I beta; myosin IC	7
1435860_at	solute carrier family 5 (sodium-dependent vitamin transporter), member 6	7
1426230_at	sphingosine kinase 2	11
1451573_a_at	syntaxin 4A (placental)	11
1423888_at	TEL2, telomere maintenance 2, homolog (S. cerevisiae)	7
1424175_at	thyrotroph embryonic factor	7
1454810_s_at	vacuolar protein sorting 8 homolog (S. cerevisiae)	11
1444307_at	--	11

CLUSTER 9		
AFFYMETRIX_3PRIME_IVT_ID	Name	Peak phase
1454617_at	arrestin domain containing 3	15
1419393_at	ATP-binding cassette, sub-family G (WHITE), member 5	15
1423999_at	c-abl oncogene 1, receptor tyrosine kinase	15
1455082_at	Casitas B-lineage lymphoma b	19
1426332_a_at	claudin 3	15
1418714_at	dual specificity phosphatase 8	15
1436866_at	ephrin A5	15
1419816_s_at	ERBB receptor feedback inhibitor 1	15
1422684_a_at	exocyst complex component 4	15
1422686_s_at	exocyst complex component 4	15
1459827_x_at	Hermansky-Pudlak syndrome 1 homolog (human)	19
1433634_at	interferon regulatory factor 2 binding protein 2	15
1417409_at	Jun oncogene	15
1448694_at	Jun oncogene	15
1432543_a_at	Kruppel-like factor 13	15
1433538_at	MARVEL (membrane-associating) domain containing 1	15
1426979_at	MLX interacting protein	15
1434384_at	nuclear receptor interacting protein 1	15
1454862_at	pleckstrin homology-like domain, family B, member 2	19
1434496_at	polo-like kinase 3 (Drosophila)	15
1418414_at	potassium voltage-gated channel, subfamily H (eag-related), member 1	15

CLUSTER 9 (continued)		
AFFYMETRIX_3PRIME_IVT_ID	Name	Peak phase
1425966_x_at	predicted gene 5928; predicted gene 12617; predicted gene 4802; similar to ribosomal protein S27a; predicted gene 13215; predicted gene 6111; predicted gene 7808; predicted gene 6014; predicted gene 8317; ubiquitin C; ubiquitin B; similar to fusion protein: ubiquitin (bases 43_513); ribosomal protein S27a (bases 217_532); similar to ubiquitin B; predicted gene 8649; ribosomal protein S27A; predicted gene 11517; predicted gene 11808; predicted gene 8430; RIKEN cDNA 2810422J05 gene; similar to Ubc protein; predicted gene 13815; ubiquitin A-52 residue ribosomal protein fusion product 1; predicted gene 8797; predicted gene 1821; predicted gene 11759; predicted gene 5239; predicted gene 6438	15
1432827_x_at	predicted gene 5928; predicted gene 12617; predicted gene 4802; similar to ribosomal protein S27a; predicted gene 13215; predicted gene 6111; predicted gene 7808; predicted gene 6014; predicted gene 8317; ubiquitin C; ubiquitin B; similar to fusion protein: ubiquitin (bases 43_513); ribosomal protein S27a (bases 217_532); similar to ubiquitin B; predicted gene 8649; ribosomal protein S27A; predicted gene 11517; predicted gene 11808; predicted gene 8430; RIKEN cDNA 2810422J05 gene; similar to Ubc protein; predicted gene 13815; ubiquitin A-52 residue ribosomal protein fusion product 1; predicted gene 8797; predicted gene 1821; predicted gene 11759; predicted gene 5239; predicted gene 6438	15
1437666_x_at	predicted gene 5928; predicted gene 12617; predicted gene 4802; similar to ribosomal protein S27a; predicted gene 13215; predicted gene 6111; predicted gene 7808; predicted gene 6014; predicted gene 8317; ubiquitin C; ubiquitin B; similar to fusion protein: ubiquitin (bases 43_513); ribosomal protein S27a (bases 217_532); similar to ubiquitin B; predicted gene 8649; ribosomal protein S27A; predicted gene 11517; predicted gene 11808; predicted gene 8430; RIKEN cDNA 2810422J05 gene; similar to Ubc protein; predicted gene 13815; ubiquitin A-52 residue ribosomal protein fusion product 1; predicted gene 8797; predicted gene 1821; predicted gene 11759; predicted gene 5239; predicted gene 6438	15
1452127_a_at	protein tyrosine phosphatase, non-receptor type 13	19
1429313_at	receptor tyrosine kinase-like orphan receptor 1	15
1428368_at	Rho GTPase activating protein 21	19
1447982_at	RIKEN cDNA 1110008P14 gene	15
1423306_at	RIKEN cDNA 2010002N04 gene	15
1430003_at	RIKEN cDNA 6330412A17 gene	15
1445913_at	RIKEN cDNA A730090H04 gene	15
1416041_at	serum/glucocorticoid regulated kinase 1	15
1419137_at	SH3/ankyrin domain gene 3	15
1434530_at	similar to DOC4; odd Oz/ten-m homolog 4 (Drosophila)	15
1448918_at	solute carrier organic anion transporter family, member 3a1	15
1422821_s_at	StAR-related lipid transfer (START) domain containing 5	19
1434225_at	SWA-70 protein	19
1438206_a_at	SYS1 Golgi-localized integral membrane protein homolog (S. cerevisiae)	19
1418559_at	testicular serine protease 1	15
1421943_at	transforming growth factor alpha	15
1417611_at	transmembrane protein 37	19
1460451_at	transmembrane protein 52	15
1428884_at	transmembrane protein 57	19
1417168_a_at	ubiquitin specific peptidase 2	15
1417169_at	ubiquitin specific peptidase 2	15
1457674_at	vacuolar protein sorting 13A (yeast)	19
1441069_at	zinc finger, DHHC domain containing 23	15

Supplementary information legends

Gremlin-2 is a BMP antagonist that is regulated by the circadian clock

Ching-Yan Chloé Yeung[1,2], Nicole Gossan[1,2], Yinhui Lu[1,2], Alun Hughes[2], James J. Hensman[3], Monika L. Bayer[4], Michael Kjær[4], Karl E. Kadler[1,2]* and Qing-Jun Meng[1,2]*

[1]Wellcome Trust Centre for Cell-Matrix Research, [2]Faculty of Life Sciences, University of Manchester, Oxford Road, Manchester M13 9PT, United Kingdom,

[3]Department of Computer Science, University of Sheffield, Sheffield S1 4DP, United Kingdom,

[4]Institute of Sports Medicine, Department of Orthopedic Surgery M, Bispebjerg Hospital, and Center for Healthy Aging, Faculty of Health Sciences, University of Copenhagen, 2400 Copenhagen, Denmark

Figure S1. Determination of local false discovery rates (lfdr) for tendon circadian time-course microarray data. Lfdr was calculated from *p*-values (reported by Circwave software) using fdrtool. (A) Histogram of *p*-values (black line) and the fit of the two-component model (colored lines). (B) Corresponding cumulative density. (C) Calculation of local false discovery rates (both tail- and density-based). *q* values were extracted from fdrtool and used to assign cut-off point for 'circadian' expression.

Figure S2. Clustering of tendon circadian genes. Rhythmic genes were organized into nine clusters, based on the time of peak-expression (shown below each pane). Each cluster shows normalized log-expression levels (y-axis) marked as points connected by a thick colored line over time (x-axis), which represents the inferred cluster-wise periodic function, with two standard deviations marked by the shaded area. DD = hours in dark/dark cycle; CT = circadian time. Grey bars represent subjective day; black bars represent subjective night. A complete list of the genes from each cluster can be found in Supplementary Table 2.

Figure S3. Phase distribution analysis of circadian expressed genes in tendon. Phase distribution of circadian genes, based on the time point at which maximum expression was detected using microarrays. Numbers in brackets indicate number of probes.

Figure S4. Full length western blots for Figure 3C.

Figure S5. Full length western blots for Figure 4A.

Supporting Tables

Supplementary Table 1. Primers used for qPCR.

Supplementary Table 2. List of rhythmic genes in tendon organized into clusters based on peak expression time.

Supplementary Video. Real-time bioluminescence microscopy of dissected Achilles tendon from *PER2::Luc* mice.
